# The expression of cuproptosis-related genes in hepatocellular carcinoma and their relationships with prognosis

**DOI:** 10.3389/fonc.2022.992468

**Published:** 2022-10-14

**Authors:** Xueying Zhao, Jin Chen, Shangqi Yin, Jingren Shi, Mei Zheng, Chaonan He, Huan Meng, Ying Han, Jinyu Han, Jingjing Guo, Zhengrong Yuan, Yajie Wang

**Affiliations:** ^1^ College of Biological Sciences and Technology, Beijing Forestry University, Beijing, China; ^2^ Department of Clinical Laboratory, Beijing Ditan Hospital, Capital Medical University, Beijing, China

**Keywords:** cuproptosis, hepatocellular carcinoma, prognosis, TCGA, bioinformatics

## Abstract

**Background:**

The mechanism of cuproptosis has recently been reported in lipoylated proteins of the tricarboxylic acid (TCA) cycle. Besides, the role of copper was previously recognized in cancer progression. We evaluated the prognostic value of cuproptosis-related gene expression in hepatocellular carcinoma (HCC).

**Methods:**

Remarkable genes were selected both in differential expression analysis and Kaplan-Meier survival analysis from ninety-six cuproptosis-related genes using The Cancer Genome Atlas (TCGA) database. The relationships between clinical characteristics and gene expression were performed with Wilcoxon signed-rank test, Kruskal-Wallis test, and logistic regression. Clinicopathologic factors correlated with overall survival in HCCs conducting univariate and multivariate Cox regression analysis. Gene Expression Profiling Interactive Analysis 2 (GEPIA2) and Human Protein Atlas (HPA) databases were utilized to verify the results. Furthermore, Gene Set Enrichment Analysis (GSEA) identified the potential key pathways that dominate cuproptosis in HCC.

**Results:**

Elevated *ATP7A*, *SLC25A3*, *SCO2*, *COA6*, *TMEM199*, *ATP6AP1*, *LIPT1*, *DLAT*, *PDHA1*, *MTF1*, *ACP1*, *FDX2*, *NUBP2*, *CIAPIN1*, *ISCA2* and *NDOR1* expression, as well as declined *AOC1*, *FDX1*, *MT-CO1*, and *ACO1* expression were significantly emerged in HCC tumor tissues and were significantly associated with HCCs poor survival. The expressions of screened cuproptosis-related genes were prominently related to clinical features. GSEA analysis reported many key signaling pathways (such as natural killer cell mediated cytotoxicity, TCA cycle, glutathione metabolism, ATP-binding cassette (ABC) transporters, Notch signaling pathway, ErbB signaling pathway, and metabolism of xenobiotics by cytochrome p450) were differentially enriched in HCCs with varying degrees of cuproptosis-related genes expression.

**Conclusions:**

The twenty cuproptosis-related genes might be utilized as new candidate prognostic biomarkers for HCC.

## Introduction

The incidence and mortality of liver cancer rank the sixth and third in the top 10 most common cancers in 2020 respectively (1), and the requirement of effective treatment and diagnostic strategies for liver cancer have been recognized to prevent recurrence, complexity, invasive metastasis, and advanced diagnosis issue. Although some serum biomarkers are well constructed as selection tools for liver cancer treatment, it is still urgent to develop specific biomarkers that evaluate the specific prognosis and predictability of treatment response for improving the detection of early or very early liver cancer (2). Liver cancer is divided into primary liver cancer and metastatic liver cancer and that hepatocellular carcinoma (HCC) is the primary malignant tumor of the liver. Hereditary hemochromatosis, hepatitis C virus, and hepatitis B virus could directly create HCC in the absence of cirrhosis, whereas HCC patients with other underlying liver diseases (such as primary biliary cirrhosis, autoimmune hepatitis, nonalcoholic steatohepatitis, Wilson disease, and alpha 1-antitrypsin deficiency) often suffer from cirrhosis, so treating the potential liver diseases and reducing the progression of cirrhosis may decline the incidence of HCC (3). Currently, treatments for HCC include chemotherapy, immunotherapy, pharmacotherapy with natural compounds, and nanotechnology therapy, the latter two of which contribute to the reduction of systemic toxicity and side effects in patients for better treatment effects (4).

Cell death has been divided into accidental cell death and programmed cell death (PCD), and PCD was subsequently redefined as regulatory cell death (RCD) (5). At present, there are dozens of RCD, among which apoptosis, necroptosis, pyroptosis, and ferroptosis are extensively researched (6). However, cuproptosis, a cell death mechanism that relies on mitochondrial respiration in human cells, is different from the previous cell death mechanisms (7). Copper can induce various forms of cell death including apoptosis and autophagy, which mainly depend on the mechanisms of antiangiogenesis, proteasome inhibition, and reactive oxygen species accumulation (8). Furthermore, copper is essential in all kinds of life processes for an important auxiliary role based on a homeostasis state, while unbalanced copper homeostasis could affect tumor growth and even cause irreversible damage to cells. Copper homeostasis disorder exists in many malignancies, with simultaneously elevated copper levels that in serum and tissues are associated with cancer progression. It has been shown that higher copper levels in serum may be associated with poorer survival in liver cancer (9). Copper chelators and copper ionophores have excellent anticancer activity, as well as copper coordination compounds have also made great progress in cancer therapy, so copper-targeted anticancer drugs have broad application prospects (10). Recently, studies of cuproptosis-related genes as potential biomarkers in tumors have been confirmed in many other cancers, such as skin cutaneous melanoma (SKCM) (11), clear cell renal cell carcinoma (ccRCC) (12), bladder cancer (BLCA) (13), triple-negative breast cancer (TNBC) (14), lung cancer (15), and head and neck squamous cell carcinoma (HNSC) (16).

The distribution of copper in organisms requires a variety of biological ligands and proteins. Copper transport protein 1 (CTR1, encoded by *SLC31A1*) allows copper to enter the cell, thereby distributing copper to different targets through metal chaperones in the cytoplasmic region of the cell. Copper chaperone for superoxide dismutase (CCS) delivers copper to superoxide dismutase 1 (SOD1), antioxidant 1 copper chaperone (ATOX1) transports copper to copper-transporting p-type adenosine triphosphatase 1 (ATP7A) and copper-transporting p-type adenosine triphosphatase 2 (ATP7B). When the copper levels in the cytoplasm are excessive, ATP7A and ATP7B are relocated to the corresponding plasma membrane to facilitate copper export from the cell (17). Chelation of copper with metallothionein (MT) or other molecules also mitigates excessive copper in cells, which is conducive to the regulation of copper homeostasis (18). The copper enzymes in mitochondria consist of cytochrome c oxidase (CCO) and SOD1, and the CuA site maturation and CuB site formation of the holoenzyme in the intermembrane space (IMS) require functions of many assembly factors, including cytochrome c oxidase copper chaperone (COX17, COX19, and COX11), synthesis of cytochrome c oxidase1 (SCO1), synthesis of cytochrome c oxidase 2 (SCO2), cytochrome c oxidase assembly factor 6 (COA6) (19). Copper is transported into mitochondria *via* SLC25A3 protein, and there are four main types of transporters in the inner membrane of mitochondria, namely mitochondrial carrier family (MCF/SLC25), ATP binding cassette (ABC) transporter, mitochondrial pyruvate carrier (MPC) and sideroflexin (FXN) (20). Four ABC transporters are located on mitochondria, including ABCB6, ABCB7, ABCB8 and ABCB10 (21).

Copper metabolic disorders include Wilson disease, Menkes disease, hereditary ceruloplasmin deficiency, and congenital disorders of glycosylation (CDG). CDG disease is triggered by gene defect of the coiled-coil domain containing 115 (*CCDC115*), transmembrane protein 199 (*TMEM199*), ATPase H+ transporting accessory protein 1 (*ATP6AP1*), and mannose phosphate isomerase (*MPI*), and is mainly manifested by varying degrees of liver injury, possibly leading to copper metabolism disorder by affecting the glycosylation modification of ATP7B protein (22, 23). Gene screening for the diagnosis of abnormal copper metabolism in liver diseases mainly involves genetic diseases that affect copper metabolism, such as gene defects of CDG-related disease, *ABCB4* gene defect disease (progressive familial intrahepatic cholestasis type 3), Adaptor related protein complex 1 subunit sigma 1 (*AP1S1*) gene defect disease (MEDNIK syndrome) and *SLC33A1* gene defect disease (Huppke-Brendl syndrome) (24).

Cells that highly depended on mitochondrial respiration are more sensitive to cuproptosis. Elesclomol as a copper ionophore inhibits the synthesis of iron-sulfur clusters resulting in a decrease of Fe-S cluster proteins. Besides, copper combines with lipoacylated dihydrolipoamide S-acetyltransferase (DLAT) regulated by ferredoxin 1 (FDX1) to increase the insoluble oligomeric DLAT when the ionophores or transporters accumulate excessively, leading to proteotoxic stress and eventually cell death (7). Copper affects the activity and stability of CCO, the terminal enzyme of the mitochondrial respiratory chain (25), and that obstructed the transport process of copper to CCO causes many human diseases. In addition, copper chaperone COX19 in mitochondria could participate in the biogenesis of CCO in humans and regulate the dynamic balance of copper and iron in plants (26). Moreover, the experiment has shown that higher concentrations of copper will lead to copper toxicity by affecting certain complexes in the respiratory chain (27).

Proteins carrying iron-sulfur clusters are essential for the basic metabolism of all organisms. Eukaryotic iron-sulfur cluster assembly includes direct mitochondrial iron-sulfur cluster (ISC) co-assembly pathway and cytosolic iron-sulfur cluster assembly (CIA) pathway that uses active sulfur as substrate, these pathways containing protein complex (composed of cysteine desulfurase, a scaffold protein, auxiliary ISD11 protein, acyl carrier protein, frataxin, and ferredoxin), the related genes (including *NFS1*, *ISD11*, *ACP1*, *ISCU2*, *FXN*, *FDX2*, *FDXR*) and proteins of cytokine induced apoptosis inhibitor 1 (CIAPIN1), novel diflavin oxidoreductase 1 (NDOR1), nucleotide binding protein 2 (NUBP2), nucleotide binding protein 1 (NUBP1), iron-only hydrogenase-like protein 1 (IOP1), and Cytosolic iron-Sulfur assembly component 1 (CIAO1) (28). Generation of mitochondrial Fe-S proteins through the ISC mechanism requires scaffold protein ISCU2, chaperone HSP70, and glutaredoxin 5 (GLRX5) (29). The imbalance of copper homeostasis in mitochondria may destroy the maturation of mitochondrial Fe-S proteins, and Cu^+^ may affect the role of mitochondrial ISCA1/ISCA2 and GLRX5 proteins in the regulation of [4Fe-4S] cluster formation (30). It is unclear whether the reduction of iron-sulfur cluster proteins promotes cell death induced by copper, but it has been found that copper ionophore elesclomol reduces iron-sulfur cluster proteins under the regulation of FDX1 (7).

A clear potential of cuproptosis-related genes with prognostic meaning and clinical diagnosis in HCC has not yet been more comprehensively studied. Accordingly, the establishment of new biomarkers related to the copper homeostasis regulatory pathway, copper metabolism illnesses, mitochondrial respiratory, and iron-sulfur cluster proteins is crucial for the early detection and prognosis of liver cancer. The Cancer Genome Atlas (TCGA) database was used to analyze the clinical information of HCCs and the mRNA expression difference in normal and HCC tissues. Differential expression analysis and survival analysis were performed to screen the genes with significant differences from ninety-six candidate cuproptosis-related genes for subsequent correlation analysis of clinical factors, univariate and multivariate Cox regression analysis. To identify the contribution of screened genes to the signaling pathway related to cuproptosis, Gene Set Enrichment Analysis (GSEA) was worked for functional enrichment analysis. Furthermore, Gene Expression Profiling Interactive Analysis 2 (GEPIA2) and Human Protein Atlas (HPA) were applied to render verification support to our outcomes from TCGA. This study was conducive to the enormous potential of cuproptosis-related genes as a new prognostic marker of HCC in the future.

## Materials and methods

**Figure 1:**
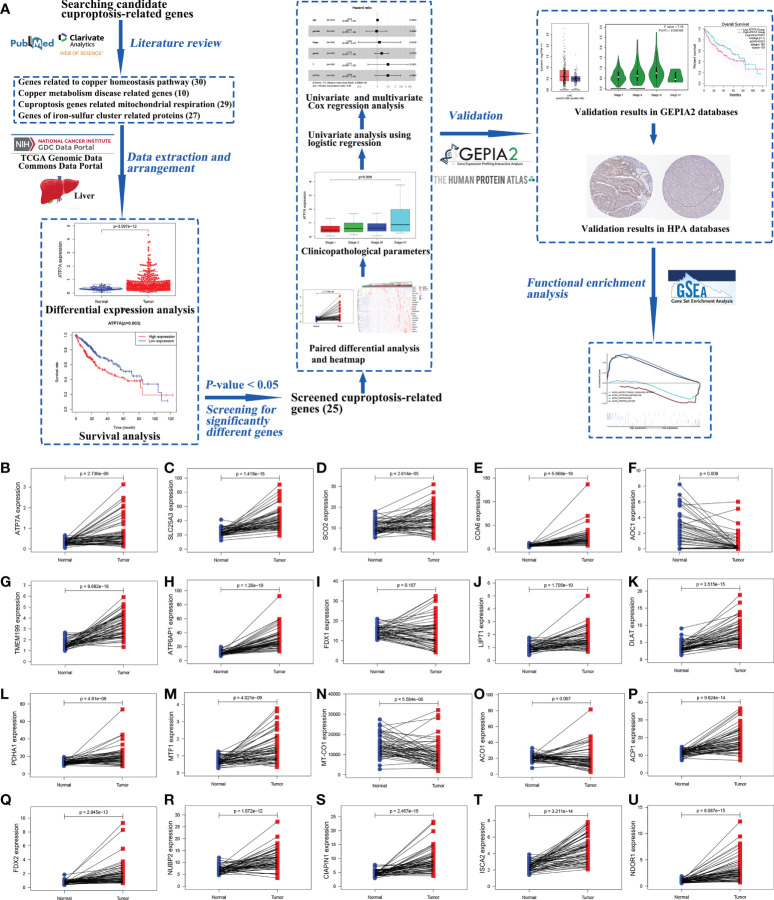
The workflow diagram of the study and the results of paired different expression analysis. The workflow diagram for exploring the expression of cuproptosis-related genes in hepatocellular carcinoma and their relationships with prognosis by bioinformatics analysis **(A)**. Statistical analysis of cuproptosis-related genes using Wilcoxon signed-rank test in 58 paired HCC samples and adjacent normal liver samples from TCGA data, including *ATP7A* **(B)**, *SLC25A3* **(C)**, *SCO2* **(D)**, *COA6* **(E)**, *AOC1* **(F)**, *TMEM199* **(G)**, *ATP6AP1* **(H)**, *FDX1* **(I)**, *LIPT1* **(J)**, *DLAT* **(K)**, *PDHA1* **(L)**, *MTF1* **(M)**, MT-*CO1* **(N)**, *ACO1* **(O)**, *ACP1* **(P)**, *FDX2* **(Q)**, *NUBP2* **(R)**, *CIAPIN1* **(S)**, *ISCA2* **(T)**, and *NDOR1* **(U)**. (ACO1, aconitase 1; ACP1, acid phosphatase 1; AOC1, amine oxidase copper containing 1; ATP6AP1, ATPase H+ transporting accessory protein 1; ATP7A, copper-transporting p-type adenosine triphosphatase 1; CIAPIN1, cytokine induced apoptosis inhibitor 1; COA6, cytochrome c oxidase assembly factor 6; DLAT, dihydrolipoamide S-acetyltransferase; FDX1, ferredoxin 1; FDX2, ferredoxin 2; GEPIA2, Gene Expression Profiling Interactive Analysis 2; GSEA, Gene Set Enrichment Analysis; HCC, hepatocellular carcinoma; HPA, Human Protein Atlas; ISCA2, iron-sulfur cluster assembly 2; LIPT1, lipoyltransferase 1; MT-CO1, mitochondrially encoded cytochrome c oxidase I; MTF1, metal regulatory transcription factor 1; NDOR1, NADPH dependent diflavin oxidoreductase 1; NUBP2, nucleotide binding protein 2; PDHA1, pyruvate dehydrogenase E1 subunit alpha 1; SCO2, synthesis of cytochrome c oxidase 2; SLC25A3, solute carrier family 25 member 3; TCGA, The Cancer Genome Atlas; TMEM199, transmembrane protein 199).

The research process was shown in a workflow diagram regarding the selection of the cuproptosis-related genes from the TCGA database and the strategy of the study (**Figure 1A**).

### Data extraction and arrangement

The transcriptome profiling with gene expression quantification as data type (465 files, a total of 407 tumor samples and 58 normal samples, Workflow Type: HTSeq- FPKM) and relevant clinical information for 418 cases in liver cancer were collected from the TCGA Genomic Data Commons (GDC) Data Portal (https://portal.gdc.cancer.gov/repository) (31). The downloaded liver cancer data in TCGA includes primary liver cancer (HCC, 374 samples) and metastatic liver cancer (cholangiocellular carcinoma, CHOL, 33 samples), and it may be instructive to consider a small number of metastatic liver cancer subset. Data were arranged in Perl (version 5.26.3) for further analyses.

### Differential expression analysis and survival analysis of candidate cuproptosis-related genes in HCC from TCGA database

The details of ninety-six candidate cuproptosis-related genes involved in the copper homeostasis regulatory pathway, copper metabolism diseases, mitochondrial respiratory, and iron-sulfur cluster proteins were exhibited in **Table S1**. All subsequent data analyses were performed by R software (version 3.6.3). Boxplots with scatters were applied to visualize the gene expression differences in normal and HCC tissues by Wilcoxon signed-rank test, and the R package “limma” and “beeswarm”. Only the samples with both survival status and survival time information could be used for survival analysis. Kaplan-Meier survival curves were evaluated using the log-rank test and drawn using the R package “survival”. As a complementary explanation, high and low gene expression was distinguished on the foundation of the median values. Only genes with a *P*-value < 0.05 both in boxplots and Kaplan-Meier survival curves could be considered as the genes with significant differences for subsequent analyses. Paired differential analysis was performed using Wilcoxon signed-rank test to demonstrate gene expression trends in HCC tissues relative to normal tissues. Heatmap was plotted in https://www.bioinformatics.com.cn (last accessed on 30 July 2022), a free online platform for data analysis and visualization.

### Correlation analysis of clinical factors and univariate and multivariate Cox regression analyses from TCGA database

The clinical data that removed missing information was conducted to visualize gene expression differences in clinical characteristics respectively. It was worth noting that the Wilcoxon signed-rank test was used between two groups and the Kruskal-Wallis test was carried out between multiple groups. The logistic regression was used in univariate analysis to reveal the associations between clinicopathological variables of different levels and gene expressions. Univariate and multivariate Cox regression analyses were implemented to identify independent prognosis risk factors. Only factors with entire information in age, gender, clinical stage, histologic grade, tumor stage (T classification), and gene expression were assessed for prognosis risk factors. Hazard ratio (HR) = *h*_1_ (*t*)/*h*_0_ (*t*) = *e^β^*. Log scale of gene expression was used log_2_ (TPM+1) for graphical aesthetics. Generally, HR > 1 indicates the gene is a risk factor, and HR < 1 indicates the gene is a protective factor. All comparisons were considered statistically significant with a *P*-value < 0.05.

### Validation in GEPIA2 and HPA databases

The GEPIA2 database (http://gepia2.cancer-pku.cn/) (32) was also performed for validating the gene expression information of screened cuproptosis-related genes for HCC in boxplots, clinical stage plots, and survival analysis. In all analyses, the gene symbol or ensemble id was input in “Gene” and liver hepatocellular carcinoma (LIHC) was selected for cancer name. “Box Plot” module: the *P*-value cutoff was 0.05, “Multiple Datasets” and “Match TCGA normal and GTEx data” were clicked, and log_2_ (TPM+1) was used for the log scale. More data from normal liver samples were increased by selecting data from the GTEx database. “Stage Plot” module: log_2_ (TPM+1) was used for the log scale, and the major stage was chosen for plotting. “Survival Analysis” module: “Overall Survival” and “Median” were clicked, and default values were used for other options. At the same time, each protein expression of screened cuproptosis-related genes with the same antibody in HCC tissues and normal liver tissues was checked by the HPA portal (https://www.proteinatlas.org/) (33).

### Identification of relevant signaling pathways of cuproptosis-related genes by GSEA

GSEA software (version 4.1.0) was operated for functional enrichment analysis of the Kyoto Encyclopedia of Genes and Genomes (KEGG) using the data from the TCGA database. The parameters of gene set parameters and run enrichment tests were set as follows. The name of each gene was selected as the expression dataset, and “c2.cp.kegg.v7.2.symbols.gmt” was chosen as the gene sets database. Setting the permutations value to default value 1000 for computing Normalized enrichment score (NES). The max size excluded larger sets and the min size excluded smaller sets were set in default values of 500 and 15, respectively. Signaling pathways with a false discovery rate (FDR) q-value < 0.05 was recognized with significant enrichment. *P*-value < 0.05 was used to select the enriched signaling pathways when q-value > 0.05. Adjusting the max size excluded larger sets to 600 or 700 when no signaling pathways have *P*-value < 0.05.

## Results

### Screened cuproptosis-related genes

Ninety-six genes that consulted in research involved in the copper homeostasis regulatory pathway, copper metabolism diseases, mitochondrial respiratory, and iron-sulfur cluster proteins were regarded as candidate cuproptosis-related genes (**Table S1**). The results of differential expression analysis and survival analysis displayed that twenty-five genes with *P*-value < 0.05 both in boxplots and Kaplan-Meier survival curves were selected for screened cuproptosis-related genes. 26.04% of candidate cuproptosis-related genes (25 out of 96 candidate genes) were differentially expressed in HCC and normal liver tissues. Nevertheless, a few genes in the twenty-five screened cuproptosis-related genes as predictors of HCC poor prognosis had been reported by the TCGA database, containing *ABCB6* (34), *CDKN2A* (35), *CDKN3* (36), *TPI1* (37), and *HSPA8* (38). Ultimately, the twenty genes including *ATP7A*, *SLC25A3*, *SCO2*, *COA6*, *AOC1*, *TMEM199*, *ATP6AP1*, *FDX1*, *LIPT1*, *DLAT*, *PDHA1*, *MTF1*, *MT-CO1*, *ACO1*, *ACP1*, *FDX2*, *NUBP2*, *CIAPIN1*, *ISCA2*, and *NDOR1* were used for eventually screened cuproptosis-related genes in later study.

### The gene expression of screened cuproptosis-related genes in HCC tissues and normal tissues from TCGA

**Figure 2:**
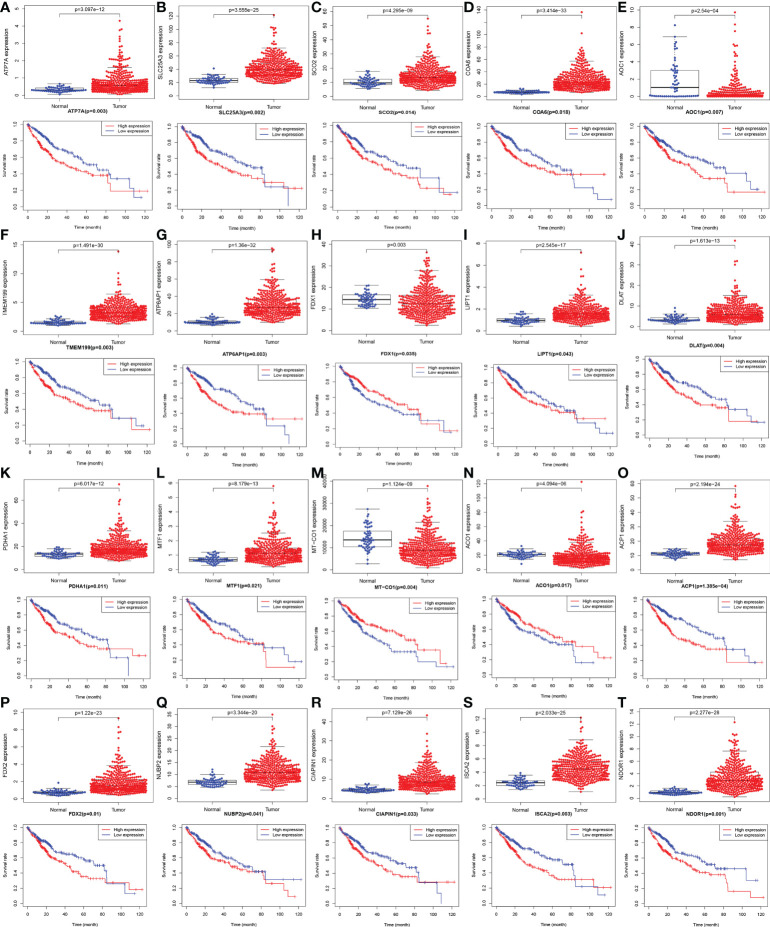
The boxplot and Kaplan-Meier survival curves with mRNA expression of screened cuproptosis-related genes in hepatocellular carcinoma (HCC) from the TCGA database. Different expression of cuproptosis-related genes between HCC tumor and normal tissues, including *ATP7A* **(A)**, *SLC25A3* **(B)**, *SCO2* **(C)**, *COA6* **(D)**, *AOC1* **(E)**, *TMEM199* **(F)**, *ATP6AP1* **(G)**, *FDX1* **(H)**, *LIPT1* **(I)**, *DLAT* **(J)**, *PDHA1* **(K)**, *MTF1* **(L)**, MT-*CO1* **(M)**, *ACO1* **(N)**, *ACP1* **(O)**, *FDX2* **(P)**, *NUBP2* **(Q)**, *CIAPIN1* **(R)**, *ISCA2* **(S)**, and *NDOR1* **(T)**. The association between survival rate and expression levels of cuproptosis-related genes in HCC, including *ATP7A* **(A)**, *SLC25A3* **(B)**, *SCO2* **(C)**, *COA6* **(D)**, *AOC1* **(E)**, *TMEM199* **(F)**, *ATP6AP1* **(G)**, *FDX1* **(H)**, *LIPT1* **(I)**, *DLAT* **(J)**, *PDHA1* **(K)**, *MTF1* **(L)**, MT-*CO1* **(M)**, *ACO1* **(N)**, *ACP1* **(O)**, *FDX2* **(P)**, *NUBP2* **(Q)**, *CIAPIN1* **(R)**, *ISCA2* **(S)**, and *NDOR1* **(T)**. (ACO1, aconitase 1; ACP1, acid phosphatase 1; AOC1, amine oxidase copper containing 1; ATP6AP1, ATPase H+ transporting accessory protein 1; ATP7A, copper-transporting p-type adenosine triphosphatase 1; CIAPIN1, cytokine induced apoptosis inhibitor 1; COA6, cytochrome c oxidase assembly factor 6; DLAT, dihydrolipoamide S-acetyltransferase; FDX1, ferredoxin 1; FDX2, ferredoxin 2; HCC, hepatocellular carcinoma; ISCA2, iron-sulfur cluster assembly 2; LIPT1, lipoyltransferase 1; MT-CO1, mitochondrially encoded cytochrome c oxidase I; MTF1, metal regulatory transcription factor 1; NDOR1, NADPH dependent diflavin oxidoreductase 1; NUBP2, nucleotide binding protein 2; PDHA1, pyruvate dehydrogenase E1 subunit alpha 1; SCO2, synthesis of cytochrome c oxidase 2; SLC25A3, solute carrier family 25 member 3; TCGA, The Cancer Genome Atlas; TMEM199, transmembrane protein 199).

**Figure 3:**
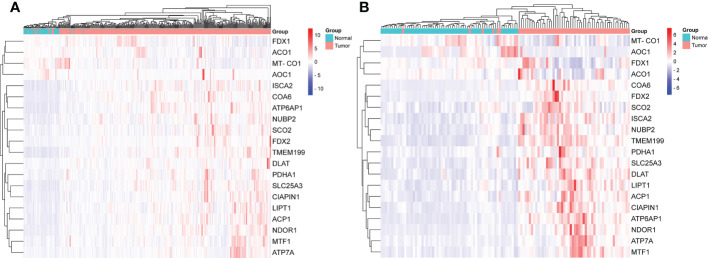
The heatmap of screened cuproptosis-related genes in normal and HCC tissues from the TCGA data. Expression levels of twenty screened cuproptosis-related genes in all samples **(A)**. Expression levels of twenty screened cuproptosis-related genes in 58 paired samples **(B)**. (ACO1, aconitase 1; ACP1, acid phosphatase 1; AOC1, amine oxidase copper containing 1; ATP6AP1, ATPase H+ transporting accessory protein 1; ATP7A, copper-transporting p-type adenosine triphosphatase 1; CIAPIN1, cytokine induced apoptosis inhibitor 1; COA6, cytochrome c oxidase assembly factor 6; DLAT, dihydrolipoamide S-acetyltransferase; FDX1, ferredoxin 1; FDX2, ferredoxin 2; HCC, hepatocellular carcinoma; ISCA2, iron-sulfur cluster assembly 2; LIPT1, lipoyltransferase 1; MT-CO1, mitochondrially encoded cytochrome c oxidase I; MTF1, metal regulatory transcription factor 1; NDOR1, NADPH dependent diflavin oxidoreductase 1; NUBP2, nucleotide binding protein 2; PDHA1, pyruvate dehydrogenase E1 subunit alpha 1; SCO2, synthesis of cytochrome c oxidase 2; SLC25A3, solute carrier family 25 member 3; TCGA, The Cancer Genome Atlas; TMEM199, transmembrane protein 199).

The gene expression levels of *AOC1* (*P* = 0.008, **Figure 1F**), *MT-CO1* (P < 0.001, **Figure 1N**), and *ACO1* (*P* = 0.007, **Figure 1O**) were significantly descended in HCC samples by comparing 58 paired HCC samples and adjacent normal liver samples in paired differential analysis, other genes were notably ascended in HCC samples (**Figure 1**). Although *FDX1* expression (*P*-value = 0.157) was not decreased statistically significant in HCC tumor tissues, the trend of *FDX1* expression was still declined by paired differential analysis (**Figure 1I**). The gene expressions of *AOC1* (*P* < 0.001, **Figure 2E**), *FDX1* (*P* = 0.003, **Figure 2H**), *MT-CO1* (*P* < 0.001, **Figure 2M**), and *ACO1* (*P* < 0.001, **Figure 2N**) in HCC tumor tissues were remarkably lower than those in normal tissues. Whereas the gene expressions of *ATP7A*, *SLC25A3*, *SCO2*, *COA6*, *TMEM199*, *ATP6AP1*, *LIPT1*, *DLAT*, *PDHA1*, *MTF1*, *ACP1*, *FDX2*, *NUBP2*, *CIAPIN1*, *ISCA2*, and *NDOR1* in HCC tissues were prominently higher than their surrounding tissues, and the differences were all statistically significant (P < 0.001, **Figure 2**). The heatmap showed the same results in all samples and paired samples from the TCGA (**Figure 3**).

### Survival analysis of screened cuproptosis-related genes from TCGA

As shown in **Figure 2**, Kaplan-Meier survival analysis showed that low expression of *FDX1* (*P* = 0.035, **Figure 2H**), *MT-CO1* (*P* = 0.004, **Figure 2M**), and *ACO1* (*P* = 0.017, **Figure 2N**) possessing worse prognosis with a poor survival rate, with contradictory high expression of *ATP7A*, *SLC25A3*, *SCO2*, *COA6*, *AOC1*, *TMEM199*, *ATP6AP1*, *LIPT1*, *DLAT*, *PDHA1*, *MTF1*, *ACP1*, *FDX2*, *NUBP2*, *CIAPIN1*, *ISCA2*, and *NDOR1* having more poorly prognosis with statistically significant (*P* < 0.05, **Figure 2**). The outcome that high expression of *AOC1* (*P* = 0.007, **Figure 2E**) that associated with poor survival rate in HCCs by survival analysis was not consistent with lower expression in HCC tumor tissues by differential expression analysis.

### Relationships between the expressions of screened cuproptosis-related genes and clinicopathological parameters from TCGA

**Figure 4:**
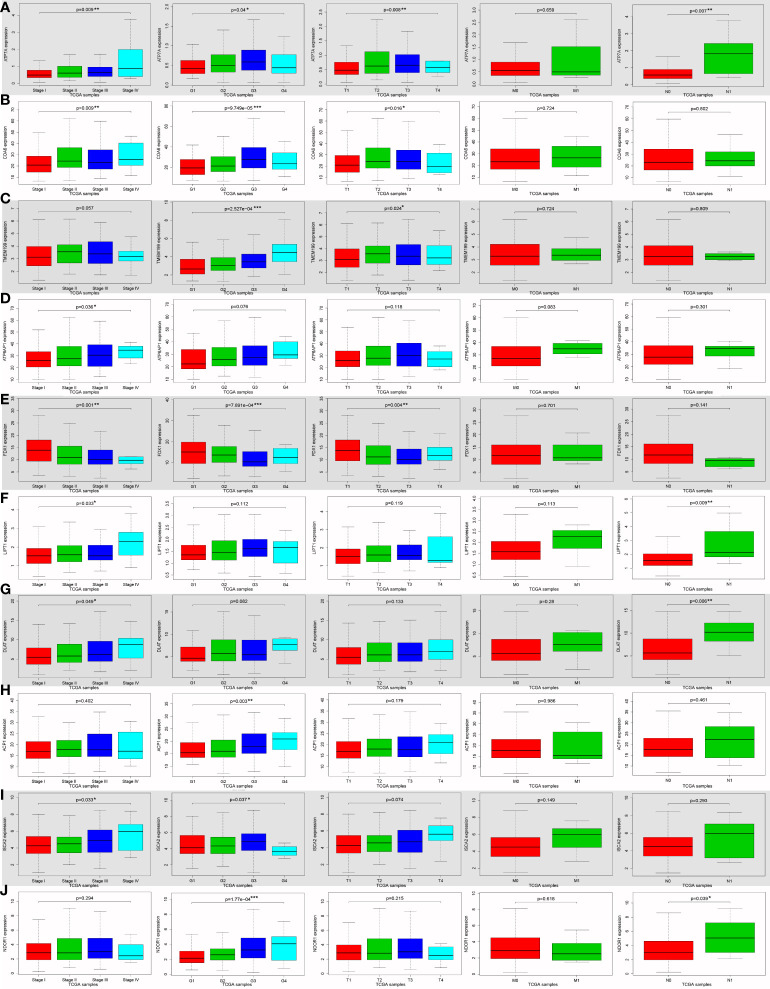
The correlation between ten cuproptosis-related gene expression levels and various clinicopathological features in hepatocellular carcinoma (HCC) patients using Wilcoxon signed-rank test or Kruskal-Wallis test from the TCGA database. The correlation between various clinicopathological features and cuproptosis-related genes expression levels in HCCs, including *ATP7A* **(A)**, *COA6* **(B)**, *TMEM199* **(C)**, *ATP6AP1* **(D)**, *FDX1* **(E)**, *LIPT1* **(F)**, *DLAT* **(G)**, *ACP1* **(H)**, *ISCA2* **(I)**, and *NDOR1* **(J)**. (ACP1, acid phosphatase 1; ATP6AP1, ATPase H+ transporting accessory protein 1; ATP7A, copper-transporting p-type adenosine triphosphatase 1; COA6, cytochrome c oxidase assembly factor 6; DLAT, dihydrolipoamide S-acetyltransferase; FDX1, ferredoxin 1; G, histologic grade; HCC, hepatocellular carcinoma; ISCA2, iron-sulfur cluster assembly 2; LIPT1, lipoyltransferase 1; M, distant metastasis; N, lymph node metastasis; NDOR1, NADPH dependent diflavin oxidoreductase 1; Stage, clinical stage; T, tumor stage; TCGA, The Cancer Genome Atlas; TMEM199, transmembrane protein 199; *P < 0.05; **P < 0.01; ***P < 0.001).

*ATP7A* (*P* = 0.009), *COA6* (*P* = 0.009), *ATP6AP1* (*P* = 0.036), *FDX1* (*P* = 0.001), *LIPT1* (*P* = 0.033), *DLAT* (*P* = 0.049) and *ISCA2* (*P* = 0.033) expression were notably associated with clinical stage (**Figures 4A, B, D-G, I**). *ATP7A* (*P* = 0.04), *COA6* (*P* < 0.001), *TMEM199* (*P* < 0.001), *FDX1* (*P* < 0.001), *ACP1* (*P* = 0.003), *ISCA2* (*P* = 0.037), *NDOR1* (*P* < 0.001), *AOC1* (*P* = 0.034) and *ACO1* (*P* = 0.01) were significantly correlated with histologic grade (**Figures 4A-C, E, H-J**; **Figures S1C, G**). *ATP7A* (*P* = 0.008), *COA6* (*P* = 0.016), *TMEM199* (*P* = 0.024) and *FDX1* (*P* = 0.004) expression were significantly related to tumor stage (T classification) (**Figures 4A-C, E**). *ATP7A* (*P* = 0.007), *LIPT1* (*P* = 0.009), *DLAT* (*P* = 0.006) and *NDOR1* (*P* = 0.039) expression were remarkably interrelated to lymph node metastasis (N classification) (**Figures 4A, F, G, J**). Univariate analysis using logistic regression revealed that gene expressions were relevant to negative prognostic clinicopathological parameters except for *SLC25A3*, *AOC1*, *PDHA1*, *MTF1*, *MT-CO1*, *NUBP2*, and *CIAPIN1* expression (**Table S2**). Elevated *SCO2*, *LIPT1*, *DLAT*, *ACP1*, *FDX2*, and *NDOR1* expression and reduced *ACO1* expression were only significantly associated with histologic grade (**Table S2**). High *ATP6AP1* expression was only notably correlated with the clinical stage (odds ratio [OR] = 10.779 for stage IV *vs*. I) (**Table S2**). Ascended *ISCA2* expression was remarkably related to both the histologic grade (OR = 1.924 for G3 *vs*. G1) and the T classification (OR = 6.661 for stage T4 *vs*. T1), whereas decreased *ISCA2* expression was significantly related to the histologic grade (OR = 0.259 for G4 *vs*. G1) (**Table S2**). Increased *ATP7A*, *COA6*, and *TMEM199* expression and diminished *FDX1* expression were all remarkably associated with clinical stage, histologic grade, and T classification (**Table S2**). These results demonstrated that patients with high *SCO2*, *ATP7A*, *COA6*, *TMEM199*, *ATP6AP1*, *LIPT1*, *DLAT*, *ACP1*, *FDX2*, *ISCA2*, and *NDOR1 *expression together with low *ACO1* and *FDX1* expression had a disposition to advanced clinical stage and histologic grade (**Table S2**).

### Hazard factors affecting patient’s survival

**Figure 5:**
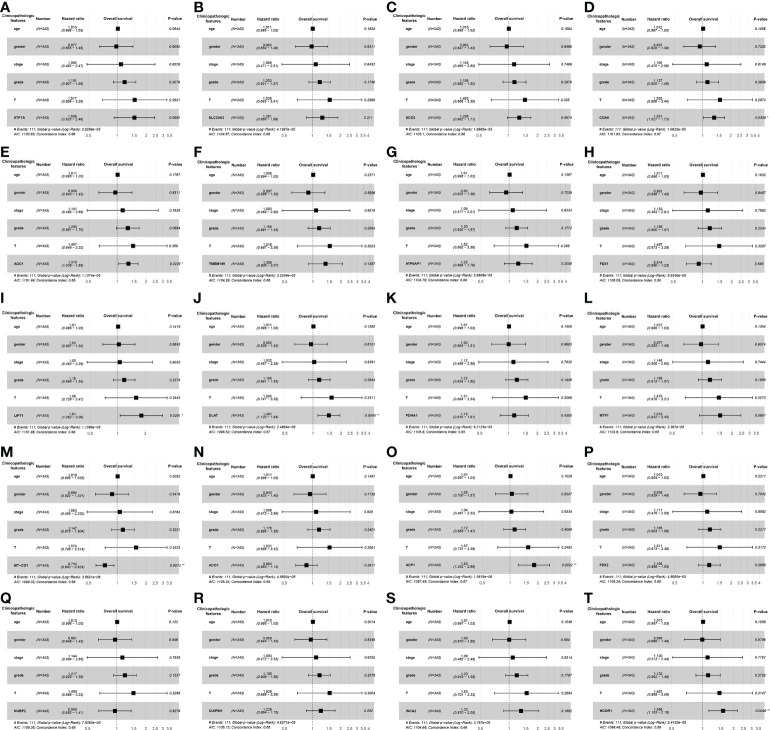
The forest plots of multivariate Cox analysis about screened cuproptosis-related gene expression levels among hepatocellular carcinoma (HCC) patients from TCGA. The forest plot was generated to show the connection between clinicopathological features and the cuproptosis-related genes expression level of HCCs, including *ATP7A* **(A)**, *SLC25A3* **(B)**, *SCO2* **(C)**, *COA6* **(D)**, *AOC1* **(E)**, *TMEM199* **(F)**, *ATP6AP1* **(G)**, *FDX1* **(H)**, *LIPT1* **(I)**, *DLAT* **(J)**, *PDHA1* **(K)**, *MTF1* **(L)**, MT-*CO1* **(M)**, *ACO1* **(N)**, *ACP1* **(O)**, *FDX2* **(P)**, *NUBP2* **(Q)**, *CIAPIN1* **(R)**, *ISCA2* **(S)**, and *NDOR1* **(T)**. (ACO1, aconitase 1; ACP1, acid phosphatase 1; AOC1, amine oxidase copper containing 1; ATP6AP1, ATPase H+ transporting accessory protein 1; ATP7A, copper-transporting p-type adenosine triphosphatase 1; CIAPIN1, cytokine induced apoptosis inhibitor 1; COA6, cytochrome c oxidase assembly factor 6; DLAT, dihydrolipoamide S-acetyltransferase; FDX1, ferredoxin 1; FDX2, ferredoxin 2; Grade, histologic grade; HCC, hepatocellular carcinoma; ISCA2, iron-sulfur cluster assembly 2; LIPT1, lipoyltransferase 1; MT-CO1, mitochondrially encoded cytochrome c oxidase I; MTF1, metal regulatory transcription factor 1; NDOR1, NADPH dependent diflavin oxidoreductase 1; NUBP2, nucleotide binding protein 2; PDHA1, pyruvate dehydrogenase E1 subunit alpha 1; SCO2, synthesis of cytochrome c oxidase 2; SLC25A3, solute carrier family 25 member 3; Stage, clinical stage; T, tumor stage; TCGA, The Cancer Genome Atlas; TMEM199, transmembrane protein 199; *P < 0.05; **P < 0.01; ***P < 0.001).

The univariate Cox regression analysis uncovered that the up-regulation of *SCO2*, *ATP7A*, *LIPT1*, *DLAT*, *MTF1*, *SLC25A3*, *AOC1*, *TMEM199*, *COA6*, *ACP1*, *FDX2*, *CIAPIN1* and *NDOR1* expression and low *MT-CO1* expression were related to poor overall survival in HCCs (**Table 1**). Other clinical variables associated with bad survival including clinical stage (hazard ratio [HR]: 1.672; 95% confidence interval [CI]: 1.359–2.056; *P* < 0.001) and T classification (HR: 1.652; 95% CI: 1.357–2.011; *P* < 0.001) (**Table 1**). In multivariate Cox analysis, high *COA6*, *AOC1*, *LIPT1*, *DLAT*, *ACP1*, and *NDOR1* expression, along with low *MT-CO1* expression maintained an independent risk factor for overall survival among HCCs (**Figure 5** and **Table 2**).

**Table 1:** Univariate Cox regression analysis for prognosis risk factors of HCC patients.

Parameter	Univariate analysis			
	HR	95%CI Low	95%CI High	*P*-value
Age	1.01	0.995	1.025	0.177
Gender	0.82	0.557	1.209	0.317
Clinical stage	1.672	1.359	2.056	< 0.001***
Histologic grade	1.121	0.868	1.446	0.382
Tumor stage	1.652	1.357	2.011	< 0.001***
*SCO2*	1.355	1.018	1.803	0.037*
*ATP7A*	1.824	1.132	2.94	0.014*
*FDX1*	0.78	0.58	1.047	0.098
*LIPT1*	2.028	1.209	3.401	0.007**
*DLAT*	1.634	1.252	2.132	< 0.001***
*PDHA1*	1.226	0.88	1.708	0.228
*MTF1*	1.657	1.03	2.664	0.037*
*SLC25A3*	1.471	1.002	2.159	0.049*
*AOC1*	1.307	1.047	1.632	0.018*
*TMEM199*	1.694	1.149	2.499	0.008**
*ATP6AP1*	1.345	0.979	1.849	0.068
*MT-CO1*	0.721	0.581	0.895	0.003**
*ACO1*	0.82	0.628	1.072	0.146
*COA6*	1.328	1.039	1.697	0.024*
*ACP1*	2.108	1.443	3.077	< 0.001***
*FDX2*	1.318	1.002	1.735	0.048*
*NUBP2*	1.203	0.828	1.746	0.332
*CIAPIN1*	1.468	1.043	2.067	0.028*
*ISCA2*	1.49	0.971	2.286	0.068
*NDOR1*	1.752	1.279	2.402	< 0.001***

ACO1, aconitase 1; ACP1, acid phosphatase 1; AOC1, amine oxidase copper containing 1; ATP6AP1, ATPase H+ transporting accessory protein 1; ATP7A, copper-transporting p-type adenosine triphosphatase 1; CI, confidence interval; CIAPIN1, cytokine induced apoptosis inhibitor 1; COA6, cytochrome c oxidase assembly factor 6; DLAT, dihydrolipoamide S-acetyltransferase; FDX1, ferredoxin 1; FDX2, ferredoxin 2; HR, hazard ratio; ISCA2, iron-sulfur cluster assembly 2; LIPT1, lipoyltransferase 1; MT-CO1, mitochondrially encoded cytochrome c oxidase I; MTF1, metal regulatory transcription factor 1; NDOR1, NADPH dependent diflavin oxidoreductase 1; NUBP2, nucleotide binding protein 2; PDHA1, pyruvate dehydrogenase E1 subunit alpha 1; SCO2, synthesis of cytochrome c oxidase 2; SLC25A3, solute carrier family 25 member 3; TMEM199, transmembrane protein 199; *P < 0.05; **P < 0.01; ***P < 0.001.

**Table 2:** Multivariate Cox regression analysis for prognosis risk factors of HCC patients.

Gene	Parameter	Multivariate analysis			
		HR	95%CI Low	95%CI High	*P*-value
*SCO2*	Age	1.01	0.995	1.025	0.188
	Gender	0.962	0.647	1.431	0.849
	Clinical stage	1.146	0.495	2.653	0.75
	Histologic grade	1.156	0.88	1.52	0.298
	Tumor stage	1.483	0.666	3.303	0.335
	*SCO2*	1.295	0.982	1.708	0.067
*ATP7A*	Age	1.013	0.998	1.029	0.094
	Gender	0.977	0.655	1.456	0.908
	Clinical stage	1.092	0.482	2.473	0.833
	Histologic grade	1.191	0.907	1.563	0.208
	Tumor stage	1.517	0.699	3.292	0.292
	*ATP7A*	1.508	0.925	2.458	0.099
*FDX1*	Age	1.011	0.996	1.026	0.16
	Gender	0.961	0.646	1.43	0.846
	Clinical stage	1.133	0.493	2.606	0.768
	Histologic grade	1.188	0.9	1.568	0.224
	Tumor stage	1.487	0.673	3.284	0.327
	*FDX1*	0.914	0.68	1.228	0.549
*LIPT1*	Age	1.011	0.996	1.027	0.142
	Gender	1.031	0.687	1.545	0.884
	Clinical stage	1.052	0.463	2.391	0.903
	Histologic grade	1.178	0.898	1.545	0.237
	Tumor stage	1.59	0.729	3.469	0.244
	*LIPT1*	1.813	1.062	3.095	0.029*
*DLAT*	Age	1.011	0.996	1.027	0.139
	Gender	0.952	0.639	1.42	0.81
	Clinical stage	1.032	0.467	2.282	0.938
	Histologic grade	1.165	0.891	1.525	0.264
	Tumor stage	1.582	0.747	3.352	0.231
	*DLAT*	1.481	1.13	1.941	0.004**
*PDHA1*	Age	1.011	0.996	1.026	0.146
	Gender	1.003	0.664	1.514	0.99
	Clinical stage	1.124	0.489	2.584	0.783
	Histologic grade	1.221	0.934	1.597	0.145
	Tumor stage	1.512	0.684	3.342	0.307
	*PDHA1*	1.147	0.816	1.611	0.43
*MTF1*	Age	1.012	0.996	1.027	0.135
	Gender	0.977	0.655	1.456	0.907
	Clinical stage	1.146	0.506	2.596	0.744
	Histologic grade	1.198	0.915	1.568	0.189
	Tumor stage	1.475	0.678	3.211	0.327
	*MTF1*	1.519	0.942	2.451	0.087
*SLC25A3*	Age	1.011	0.996	1.026	0.152
	Gender	0.983	0.659	1.465	0.931
	Clinical stage	1.088	0.471	2.515	0.843
	Histologic grade	1.203	0.921	1.572	0.175
	Tumor stage	1.538	0.693	3.413	0.29
	*SLC25A3*	1.281	0.869	1.889	0.211
*AOC1*	Age	1.011	0.995	1.026	0.177
	Gender	0.958	0.643	1.427	0.831
	Clinical stage	1.141	0.485	2.686	0.762
	Histologic grade	1.29	0.981	1.697	0.068
	Tumor stage	1.467	0.648	3.321	0.358
	*AOC1*	1.31	1.038	1.653	0.023*
*TMEM199*	Age	1.009	0.994	1.025	0.237
	Gender	0.897	0.596	1.348	0.6
	Clinical stage	1.083	0.469	2.499	0.852
	Histologic grade	1.169	0.891	1.534	0.258
	Tumor stage	1.518	0.687	3.358	0.302
	*TMEM199*	1.368	0.906	2.065	0.136
*ATP6AP1*	Age	1.011	0.996	1.027	0.137
	Gender	0.93	0.623	1.39	0.724
	Clinical stage	1.093	0.477	2.508	0.833
	Histologic grade	1.203	0.92	1.574	0.177
	Tumor stage	1.524	0.692	3.356	0.295
	*ATP6AP1*	1.249	0.888	1.756	0.201
*MT-CO1*	Age	1.01	0.995	1.025	0.205
	Gender	0.884	0.592	1.321	0.548
	Clinical stage	1.063	0.485	2.332	0.878
	Histologic grade	1.147	0.875	1.504	0.322
	Tumor stage	1.574	0.748	3.314	0.232
	*MT-CO1*	0.744	0.6	0.924	0.007**
*ACO1*	Age	1.011	0.996	1.026	0.15
	Gender	0.943	0.633	1.405	0.774
	Clinical stage	1.098	0.472	2.555	0.828
	Histologic grade	1.178	0.896	1.549	0.24
	Tumor stage	1.531	0.685	3.423	0.299
	*ACO1*	0.864	0.663	1.127	0.281
*COA6*	Age	1.012	0.997	1.027	0.106
	Gender	0.933	0.628	1.386	0.73
	Clinical stage	1.106	0.475	2.577	0.815
	Histologic grade	1.127	0.855	1.486	0.396
	Tumor stage	1.535	0.686	3.439	0.297
	*COA6*	1.331	1.027	1.726	0.031*
*ACP1*	Age	1.013	0.997	1.028	0.103
	Gender	1.048	0.7	1.567	0.821
	Clinical stage	1.035	0.461	2.324	0.933
	Histologic grade	1.122	0.855	1.473	0.407
	Tumor stage	1.574	0.731	3.387	0.246
	*ACP1*	1.826	1.242	2.685	0.002**
*FDX2*	Age	1.01	0.994	1.025	0.221
	Gender	0.938	0.629	1.399	0.754
	Clinical stage	1.111	0.476	2.594	0.808
	Histologic grade	1.185	0.903	1.555	0.222
	Tumor stage	1.511	0.673	3.39	0.317
	*FDX2*	1.166	0.888	1.53	0.269
*NUBP2*	Age	1.012	0.996	1.027	0.133
	Gender	0.961	0.646	1.431	0.846
	Clinical stage	1.144	0.494	2.651	0.753
	Histologic grade	1.217	0.929	1.594	0.154
	Tumor stage	1.49	0.669	3.315	0.329
	*NUBP2*	0.958	0.652	1.408	0.828
*CIAPIN1*	Age	1.01	0.995	1.025	0.201
	Gender	0.959	0.644	1.427	0.835
	Clinical stage	1.093	0.472	2.533	0.836
	Histologic grade	1.19	0.908	1.558	0.208
	Tumor stage	1.525	0.686	3.391	0.3
	*CIAPIN1*	1.228	0.864	1.745	0.252
*ISCA2*	Age	1.013	0.997	1.028	0.105
	Gender	1.002	0.67	1.498	0.994
	Clinical stage	1.093	0.482	2.481	0.831
	Histologic grade	1.204	0.919	1.577	0.179
	Tumor stage	1.528	0.701	3.329	0.286
	*ISCA2*	1.334	0.87	2.046	0.186
*NDOR1*	Age	1.013	0.997	1.029	0.101
	Gender	0.995	0.666	1.486	0.98
	Clinical stage	1.12	0.513	2.444	0.777
	Histologic grade	1.132	0.862	1.486	0.373
	Tumor stage	1.461	0.698	3.061	0.315
	*NDOR1*	1.586	1.153	2.182	0.005**

ACO1, aconitase 1; ACP1, acid phosphatase 1; AOC1, amine oxidase copper containing 1; ATP6AP1, ATPase H+ transporting accessory protein 1; ATP7A, copper-transporting p-type adenosine triphosphatase 1; CI, confidence interval; CIAPIN1, cytokine induced apoptosis inhibitor 1; COA6, cytochrome c oxidase assembly factor 6; DLAT, dihydrolipoamide S-acetyltransferase; FDX1, ferredoxin 1; FDX2, ferredoxin 2; HR, hazard ratio; ISCA2, iron-sulfur cluster assembly 2; LIPT1, lipoyltransferase 1; MT-CO1, mitochondrially encoded cytochrome c oxidase I; MTF1, metal regulatory transcription factor 1; NDOR1, NADPH dependent diflavin oxidoreductase 1; NUBP2, nucleotide binding protein 2; PDHA1, pyruvate dehydrogenase E1 subunit alpha 1; SCO2, synthesis of cytochrome c oxidase 2; SLC25A3, solute carrier family 25 member 3; TMEM199, transmembrane protein 199; *P < 0.05; **P < 0.01; ***P < 0.001.

### Validation results in GEPIA2 and HPA databases

GEPIA2 analysis showed that all survival curves and the gene expressions of screened cuproptosis-related genes in normal and LIHC tumor tissues were in accordant with the results from the TCGA database, which confirmed our findings at the gene level (**Figures S2** and **S3**). Compared with the results of the TCGA database, only the *COA6* and *ATP6AP1* expressions were significantly different between LIHC tumor tissues and normal tissues (**Figures S2D, E**) from the GEPIA2 database. In addition, *ATP7A* (P < 0.001), *COA6* (*P* = 0.0134), *ATP6AP1* (*P* = 0.0294), *DLAT* (*P* = 0.0484), *ACP1* (*P* = 0.0191), *NDOR1* (*P* = 0.00393), *FDX1* (*P* < 0.001), *PDHA1* (*P* = 0.02), *MTF1* (*P* = 0.0123), and *CIAPIN1* (*P* = 0.0392) expression were notably associated with clinical stage (**Figures S2A, D-G, J**; **Figures S3C, E, F, J**). More importantly, high *ATP7A*, *SLC25A3*, *SCO2*, *COA6*, *ATP6AP1*, *DLAT*, *ACP1*, *FDX2*, *ISCA2*, and *NDOR1* expression were correlated with poor overall survival in LIHCs (**Figure S2**), but only low *MT-CO1* expression (*P* = 0.0094, **Figure S3G**) was associated with poor overall survival in LIHCs. The immunohistochemistry (IHC) results in the HPA database demonstrated our results that the difference in expression between HCC and normal tissues at the protein level (**Figure S4**). However, the IHC results of SLC25A3 protein between normal and HCC tumor tissues were not existed in HPA, besides the obvious trend differences of ATP7A and ATP6AP1 expression in HCC patients were difficult to show in the HPA database.

### The correlative signaling pathways of cuproptosis-related genes in GSEA

**Figure 6:**
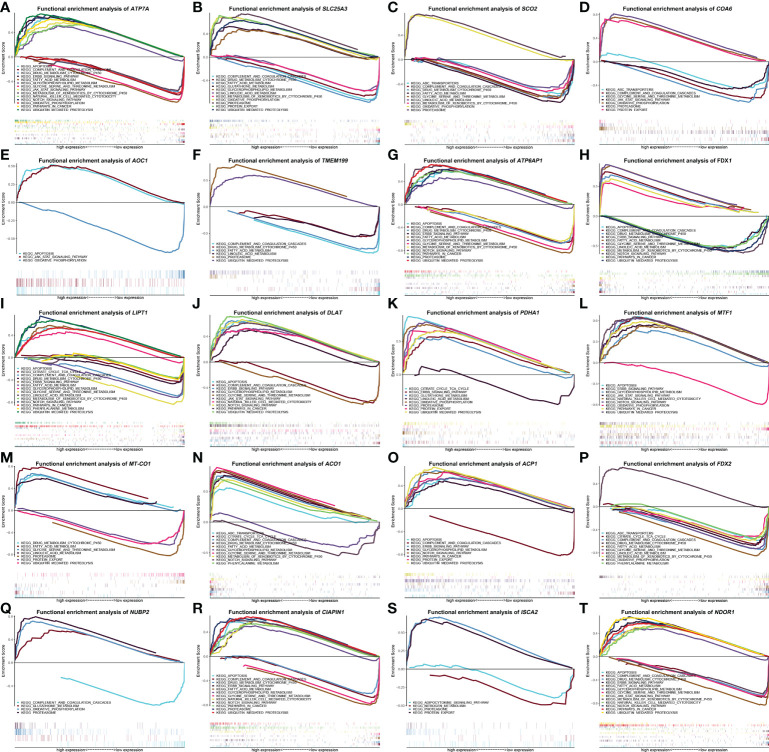
The enrichment plots of screened cuproptosis-related gene expression levels in hepatocellular carcinoma (HCC) patients from Gene Set Enrichment Analysis (GSEA). Functional enrichment analysis of cuproptosis-related genes expression in TCGA HCCs, including *ATP7A* **(A)**, *SLC25A3* **(B)**, *SCO2* **(C)**, *COA6* **(D)**, *AOC1* **(E)**, *TMEM199* **(F)**, *ATP6AP1* **(G)**, *FDX1* **(H)**, *LIPT1* **(I)**, *DLAT* **(J)**, *PDHA1* **(K)**, *MTF1* **(L)**, MT-*CO1* **(M)**, *ACO1* **(N)**, *ACP1* **(O)**, *FDX2* **(P)**, *NUBP2* **(Q)**, *CIAPIN1* **(R)**, *ISCA2* **(S)**, and *NDOR1* **(T)**. (ACO1, aconitase 1; ACP1, acid phosphatase 1; AOC1, amine oxidase copper containing 1; ATP6AP1, ATPase H+ transporting accessory protein 1; ATP7A, copper-transporting p-type adenosine triphosphatase 1; CIAPIN1, cytokine induced apoptosis inhibitor 1; COA6, cytochrome c oxidase assembly factor 6; DLAT, dihydrolipoamide S-acetyltransferase; FDX1, ferredoxin 1; FDX2, ferredoxin 2; HCC, hepatocellular carcinoma; ISCA2, iron-sulfur cluster assembly 2; LIPT1, lipoyltransferase 1; MT-CO1, mitochondrially encoded cytochrome c oxidase I; MTF1, metal regulatory transcription factor 1; NDOR1, NADPH dependent diflavin oxidoreductase 1; NUBP2, nucleotide binding protein 2; PDHA1, pyruvate dehydrogenase E1 subunit alpha 1; SCO2, synthesis of cytochrome c oxidase 2; SLC25A3, solute carrier family 25 member 3; TCGA, The Cancer Genome Atlas; TMEM199, transmembrane protein 199).

To identify signaling pathways that are distinguishingly activated in HCC, GSEA was conducted to compare the high and low gene expression datasets according to the median value. The most significantly enriched signaling pathways were chosen based on their q-value (< 0.05) or *P*-value (< 0.05). As shown in **Figure 6**, different degrees of high *ATP7A*, *ATP6AP1*, *LIPT1*, *DLAT*, *MTF1*, *ACP1*, *CIAPIN1*, and *NDOR1* expression were related to apoptosis, ubiquitin mediated proteolysis, glycerophospholipid metabolism, pathways in cancer, Notch signaling pathway, and ErbB signaling pathway. On the contrary, the apoptosis, pathways in cancer, and ErbB signaling pathway were merely enriched in low *FDX1* expression (**Figure 6H**), the glycerophospholipid metabolism was barely enriched in low *ACO1* expression (**Figure 6N**), the Notch signaling pathway was enriched in low *FDX1* and *ACO1* expression (**Figures 6H, N**), the ubiquitin mediated proteolysis was enriched in low *FDX1* and *MT-CO1* expression (**Figures 6H, M**). In addition, the pathway of proteasome demonstrated distinct degrees of high *SLC25A3*, *SCO2*, *COA6*, *TMEM199*, *ATP6AP1*, *PDHA1*, *NUBP2*, *CIAPIN1*, and *ISCA2* expression, but only showed low *MT-CO1* expression (**Figure 6M**). The pathway of protein export revealed various degrees of high *SLC25A3*, *COA6*, *PDHA1*, *ACP1*, and *ISCA2* expression, but only displayed low *MT-CO1* expression (**Figure 6M**). The janus kinase/signal transducers and activators of the transcription (JAK/STAT) signaling pathway show diverse degrees of high *ATP7A*, *AOC1*, *DLAT*, *MTF1*, and *NDOR1* expression, but only exhibited low *COA6* expression (**Figure 6D**). More importantly, natural killer cell mediated cytotoxicity was enriched in high *ATP7A*, *DLAT*, *MTF1*, *CIAPIN1*, and *NDOR1* expression. It is noteworthy that high *SLC25A3*, *PDHA1*, and *NUBP2* expression were associated with glutathione metabolism, and only high *ACO1* expression was correlated with ABC transporters and phenylalanine metabolism (**Figure 6N**), with contradictory ABC transporters, was enriched in different levels of low *SCO2*, *COA6*, and *FDX2* expression, phenylalanine metabolism was enriched in varying levels of low *LIPT1* and *FDX2* expression. The citrate cycle (tricarboxylic acid [TCA] cycle) was enriched in distinct levels of high *PDHA1* and *ACO1* expression, as well as low *LIPT1* and *FDX2* expression. Finally, gene sets of drug metabolism cytochrome p450, fatty acid metabolism, and metabolism of xenobiotics by cytochrome p450 were enriched in various levels of high *FDX1* and *ACO1* expression, along with low *ATP7A*, *SLC25A3*, *SCO2*, *ATP6AP1*, *LIPT1*, *ACP1*, *FDX2*, and *NDOR1* expression.

## Discussion

Cell death plays a key role in the reproduction and development of life. Normal cell death removes potentially abnormal and harmful cells, while the occurrence of some abnormal processes in the cell death signaling pathway will lead to organismal damage and disease, such as cancer, autoimmune diseases, and degenerative diseases (39). Cell death forms unrelated to the cell cycle are immunogenic and have the potential to confer additional anti-tumor activity to the host immunity, such as necroptosis and ferroptosis (40). It has been found that ferroptosis, autophagy, and pyroptosis were closely associated with the occurrence, development, and regression of colorectal cancer (41). In addition, pyroptosis death had a dual function of promoting and inhibiting the formation of tumor and tumor microenvironment (TME), and was involved in the occurrence and development of gastric cancer, ovarian cancer, primary liver cancer, prostate cancer, and cervical cancer (42). Similarly, in studies on the regulation and therapeutic applications of liver cancer, ferroptosis and the interaction between autophagy and apoptosis have been studied more frequently (43, 44), while there is no research related to the prognostic and therapeutic regulation biomarkers based on mRNA expression of more entire cuproptosis-related genes in HCC. Studying the expression of cuproptosis-related genes in HCC and their prognostic value will provide a foundation and new latent therapeutic targets for prognosis and clinical treatment.

In the present study, we performed differential expression analysis and survival analysis of ninety-six candidate cuproptosis-related genes involved in the copper homeostasis regulatory pathway, copper metabolism diseases, mitochondrial respiratory, and iron-sulfur cluster proteins with RNA-sequencing data of liver cancer from the TCGA database. Twenty-five candidate cuproptosis-related genes expression were significantly unequal between tumor and normal liver tissues, and they were also significantly correlated with poor survival in HCCs. Previous studies have found that *ABCB6* (34), *CDKN2A* (35), *CDKN3* (36), and *TPI1* (37) (catalyzing the conversion of dihydroxyacetone phosphate (DHAP) and D-type glyceraldehyde 3-phosphate (G3P)), as well as *HSPA8* (38), were potential targets for the treatment of liver cancer, which is consistent with our analysis results. Subsequently, patients with elevated *SCO2*, *LIPT1*, *DLAT*, *ACP1*, *FDX2*, *NDOR1*, *ATP6AP1*, *ISCA2*, *ATP7A*, *COA6*, and *TMEM199* expression together with reduced *ACO1* and *FDX1* expression had a disposition to advanced clinical stage and histologic grade by univariate analysis using logistic regression. In multivariate Cox analysis, increased *LIPT1*, *DLAT*, *AOC1*, *COA6*, *ACP1*, and *NDOR1* expression, along with declined *MT-CO1* expression maintained an independent risk factor for overall survival among HCCs, respectively. In addition, the *LIPT1* and *ISCA2* expressions were not notably associated with the clinical stage in HCCs by the GEPIA2 database against the TCGA database, whereas the *PDHA1*, *MTF1*, *ACP1*, *CIAPIN1*, and *NDOR1* expressions were remarkably related to the clinical stage in HCCs in GEPIA2 database only. GEPIA2 database showed that more screened cuproptosis-related genes were prominently correlated with negative survival in HCCs. Furthermore, the protein expressions of screened cuproptosis-related genes in HCC patients and normal liver samples were testified by the HPA database.

The functions of *FDX1*, *LIPT1*, *DLAT*, *PDHA1*, *MTF1*, *MT-CO1*, *ACO1*, *SCO2*, *ATP7A*, *SLC25A3*, *AOC1*, *COA6*, *TMEM199*, *ATP6AP1*, *ACP1*, *FDX2*, *NUBP2*, *CIAPIN1*, *ISCA2*, and *NDOR1* were investigated in HCC from TCGA data by GSEA. Some signaling pathways (such as natural killer cell mediated cytotoxicity, oxidative phosphorylation, TCA cycle, glutathione metabolism, protein export, ABC transporters, ubiquitin mediated proteolysis, apoptosis, pathways in cancer, Notch signaling pathway, JAK/STAT signaling pathway, ErbB signaling pathway, metabolism of xenobiotics by cytochrome p450, as well as complement and coagulation cascades) that differentially enriched in high or low expression of twenty screened cuproptosis-related genes were more comprehensively reported in the present study. These results suggested that *FDX1*, *LIPT1*, *DLAT*, *PDHA1*, *MTF1*, *MT-CO1*, *ACO1*, *SCO2*, *ATP7A*, *SLC25A3*, *AOC1*, *COA6*, *TMEM199*, *ATP6AP1*, *ACP1*, *FDX2*, *NUBP2*, *CIAPIN1*, *ISCA2*, and *NDOR1* may serve as prognostic markers and potential therapeutic targets in HCC, which were not summarized in other bioinformatic analysis research of HCC.

Strict regulation of copper homeostasis not only ensures copper protein biosynthesis, but also limits the occurrence and toxicity of oxidative stress, and copper as a limiting factor in cancer development processes including growth, angiogenesis, and metastasis has attracted extensive attention (45). Chronic liver injury spontaneously developed into liver cancer for copper accumulation and MT protein induction in the liver of Long Evans Cinnamon (LEC) rats (exhibiting human Wilson’s disease), with consistently, copper accumulation may inhibit the growth of surrounding liver cells, whereas the cells in injured liver tissues escape the effects of copper toxicity by increasing MT protein induction and decreasing copper accumulation to achieve proliferation (46). Copper metabolism, as a new therapeutic biomarker and a potential target for radionuclide therapy in HCC, has potential applications in molecular imaging and targeted therapy of HCC, besides copper chelators or human copper transporter 1 (hCTR1) specific short-interference RNA that blocking copper absorption could be used to inhibit the growth of HCC in the therapy of copper regulation (47). In our study, we selected a total of 40 genes related to copper homeostasis regulation and copper metabolism diseases as candidate cuproptosis-related genes. The results showed that the expressions of *SCO2*, *ATP7A*, *SLC25A3*, *AOC1*, *COA6*, *ABCB6*, *TMEM199*, and *ATP6AP* were all differently expressed in normal and HCC tissues and were significantly correlated with the low survival rate of HCC patients. It was puzzling that the survival time of patients with low *AOC1* expression was superior to high *AOC1* expression, which is contrary to lower *AOC1* expression in HCC tissues from the results of TCGA and GEPIA databases. Furthermore, the data for *AOC1* expression was very discrete in TCGA and GEPIA databases. However, upregulated *AOC1* expression in HCC tissues was associated with a poor prognosis in the study that used a large number of clinical samples (Tumor tissues and adjacent-normal tissues of 85 patients from the hospital) and highly aggregated data (48). Therefore, the more samples between tumor *vs*. normal tissues from the same patient and the more concentrated data distribution, the more reliable the results.

Recently, Copper death, a form of cell death that targets lipoylated proteins in the TCA cycle (7), provides a new entry point for cancer research. Mitochondrial respiration is highly correlated with cuproptosis. Tumor cells prefer aerobic glycolysis over oxidative phosphorylation (OXPHOS), besides, inhibition of glycolysis by gluconeogenesis and improvement of the TME could impede HCC progression (49). Copper depletion in mitochondria switches cellular metabolism from cellular respiration to glycolysis (50). The Warburg effect in HCC is mainly characterized by increased glucose uptake, elevated glycolysis, restricted mitochondrial OXPHOS, heightened glutamine catabolism, and enhanced pentose phosphate pathway in HCC cells, and is closely associated with HCC cell proliferation, migration, invasion, apoptosis, immune escape, chemotherapy suppression and treatment failure (51). Thus, the role of copper in hepatocarcinogenesis indicated that cuproptosis may be intimately associated with alleviation of mitochondrial dysfunction and reduction of glycolysis in HCC patients, providing an idea into the discovery of novel therapies related to cuproptosis for liver cancer. Other studies have also investigated the prognostic prediction of cuproptosis-related genes in HCC in terms of LncRNA signature and TME (52, 53). Twenty-nine genes associated with the TCA cycle or mitochondrial respiration were analyzed as candidate cuproptosis-related genes in this study. Reduced *FDX1*, *MT-CO1*, and *ACO1* expression have existed in HCC tissues, but *LIPT1*, *DLAT*, *PDHA1*, *MTF1*, *CDKN2A*, *CDKN3*, and *TPI1* expression were higher in HCC tissues than in normal liver tissues. Besides, lessened *FDX1*, *MT-CO1*, and *ACO1* expression along with up-regulated *LIPT1*, *DLAT*, *PDHA1*, *MTF1*, *CDKN2A*, *CDKN3*, and *TPI1* expression were significantly associated with poor survival in HCC.

A decrease in iron-sulfur cluster proteins is associated with cuproptosis, but the underlying relationship between iron-sulfur cluster proteins in the diagnosis and prognosis of HCC remains unclear. Considering the combination of targeting Fe-S cluster and other cell killing modalities, may provide new possibilities for cancer diagnosis, prognosis, and treatment (54). Significantly elevated iron-sulfur cluster proteins (ACP1, FDX2, NUBP2, CIAPIN1, ISCA2, NDOR1, and HSPA8) of HCC tissues in our study would be new potential targets of therapeutic drugs for HCC.

## Conclusion

Our research found the affinitive relationship between twenty cuproptosis-related genes expression (*FDX1*, *LIPT1*, *DLAT*, *PDHA1*, *MTF1*, *MT-CO1*, *ACO1*, *SCO2*, *ATP7A*, *SLC25A3*, *AOC1*, *COA6*, *TMEM199*, *ATP6AP1*, *ACP1*, *FDX2*, *NUBP2*, *CIAPIN1*, *ISCA2*, and *NDOR1*) and the prognosis of HCC. Moreover, the natural killer cell mediated cytotoxicity, oxidative phosphorylation, TCA cycle, glutathione metabolism, protein export, ABC transporters, ubiquitin mediated proteolysis, apoptosis, pathways in cancer, Notch signaling pathway, JAK/STAT signaling pathway, ErbB signaling pathway, metabolism of xenobiotics by cytochrome p450, and complement and coagulation cascades may be the key pathways controlled by cuproptosis-related genes in HCC. Therefore, cuproptosis-related genes may become important markers and new targets for early diagnosis, precise treatment, and prognostic assessment of HCC.

## Data availability statement

The original contributions presented in the study are included in the article/**Supplementary Material**. Further inquiries can be directed to the corresponding authors.

## Author contributions

Formal analysis, data curation, writing - original draft, validation, visualization, data analysis, and writing - review and editing were performed by XZ. Investigation, data analysis, writing - review and editing were performed by JC. Project administration and resources were performed by SY, JS, MZ, CH, HM, YH, JH, and JG. Supervision and writing - review and editing were performed by ZY and YW. All authors contributed to the article and approved the submitted version.

## Funding

This work was funded by the Beijing High-level Public Health Technical Personnel Construction Project (Grant NO. 2022-2-014), and Wu Jieping Medical Foundation of China (Grant NO. 320.6750.19089-103 and Grant NO. 320.6750.19089-75).

## Acknowledgments

Thanks for the support of the Beijing High-level Public Health Technical Personnel Construction Project (Grant NO. 2022-2-014) and Wu Jieping Medical Foundation of China (Grant NO. 320.6750.19089-103 and Grant NO. 320.6750.19089-75).

## Conflict of interest

The authors declare that the research was conducted in the absence of any commercial or financial relationships that could be construed as a potential conflict of interest.

## Publisher’s note

All claims expressed in this article are solely those of the authors and do not necessarily represent those of their affiliated organizations, or those of the publisher, the editors and the reviewers. Any product that may be evaluated in this article, or claim that may be made by its manufacturer, is not guaranteed or endorsed by the publisher.
