# Supplementary material for: The expression of cuproptosis-related genes in hepatocellular carcinoma and their relationships with prognosis
Source: Front Oncol. 2022 Oct 14;12:992468. doi: 10.3389/fonc.2022.992468 (PMC9614267; doi:10.3389/fonc.2022.992468)
Supplement: Supplementary file 1 [file DataSheet_1.docx]

**Supplementary Table 1.** Information of ninety-six candidate cuproptosis-related genes and their analytical results in HCC from the TCGA database

| Gene classification | Number | Gene symbol | Description | Ensembl ID | Differential expression analysis in normal and HCC tissues | Gene expression trend in HCC tissues | | Survival analysis |
| --- | --- | --- | --- | --- | --- | --- | --- | --- |
| Genes related to copper homeostasis pathway | 1 | *SCO2* | Synthesis Of Cytochrome C Oxidase 2 | ENSG00000284194 | ***P* < 0.001***** | | Increase | ***P* = 0.014*** |
|  | 2 | *ATP7A* | Copper-transporting P-type Adenosine Triphosphatase 1 | ENSG00000165240 | ***P* < 0.001***** | | Increase | ***P* = 0.003**** |
|  | 5 | *SLC25A3* | Solute Carrier Family 25 Member 3 | ENSG00000075415 | ***P* < 0.001***** | | Increase | ***P* = 0.002**** |
|  | 6 | *AOC1* | Amine Oxidase Copper Containing 1 | ENSG00000002726 | ***P* < 0.001***** | | Decline | ***P* = 0.007**** |
|  | 3 | *COA6* | Cytochrome C Oxidase Assembly Factor 6 | ENSG00000168275 | ***P* < 0.001***** | | Increase | ***P* = 0.018*** |
|  | 4 | *ABCB6* | ATP Binding Cassette Subfamily B Member 6 (Langereis Blood Group) | ENSG00000115657 | ***P* < 0.001***** | | Increase | ***P* = 0.007**** |
|  | 7 | *CP* | Ceruloplasmin | ENSG00000047457 | ***P* < 0.001***** | | Decline | *P* = 0.185 |
|  | 8 | *SLC31A1* | Solute Carrier Family 31 Member 1 | ENSG00000136868 | ***P* < 0.001***** | | Decline | *P* = 0.05 |
|  | 9 | *SLC31A2* | Solute Carrier Family 31 Member 2 | ENSG00000136867 | ***P* < 0.001***** | | Decline | *P* = 0.322 |
|  | 10 | *ATOX1* | Antioxidant 1 Copper Chaperone | ENSG00000177556 | ***P* < 0.001***** | | Increase | *P* = 0.239 |
|  | 11 | *COX17* | Cytochrome C Oxidase Copper Chaperone COX17 | ENSG00000138495 | ***P* < 0.001***** | | Increase | *P* = 0.147 |
|  | 12 | *COX11* | Cytochrome C Oxidase Copper Chaperone COX11 | ENSG00000166260 | ***P* < 0.001***** | | Increase | *P* = 0.327 |
|  | 13 | *CCS* | Copper Chaperone For Superoxide Dismutase | ENSG00000173992 | *P* = 0.778 | | Uncertain |  |
|  | 14 | *SCO1* | Synthesis Of Cytochrome C Oxidase 1 | ENSG00000133028 | ***P* = 0.033*** | | Increase | *P* = 0.73 |
|  | 15 | *ATP7B* | ATPase Copper Transporting Beta | ENSG00000123191 | *P* = 0.328 | | Uncertain |  |
|  | 16 | *SOD1* | Superoxide Dismutase 1 | ENSG00000142168 | ***P* = 0.003**** | | Decline | *P* = 0.234 |
|  | 17 | *SOD2* | Superoxide Dismutase 2 | ENSG00000112096 | *P* = 0.45 | | Uncertain |  |
|  | 18 | *SOD3* | Superoxide Dismutase 3 | ENSG00000109610 | *P* = 0.065 | | Uncertain |  |
|  | 19 | *MT1A* | Metallothionein 1A | ENSG00000205362 | ***P* < 0.001***** | | Decline | *P* = 0.56 |
|  | 20 | *MT2A* | Metallothionein 2A | ENSG00000125148 | ***P* < 0.001***** | | Decline | *P* = 0.376 |
|  | 21 | *MT3* | Metallothionein 3 | ENSG00000087250 | ***P* < 0.001***** | | Increase | *P* = 0.26 |
|  | 22 | *ABCB8* | ATP Binding Cassette Subfamily B Member 8 | ENSG00000197150 | ***P* < 0.001***** | | Increase | *P* = 0.688 |
|  | 23 | *ABCB10* | ATP Binding Cassette Subfamily B Member 10 | ENSG00000135776 | ***P* < 0.001***** | | Increase | *P* = 0.962 |
|  | 24 | *ABCB7* | ATP Binding Cassette Subfamily B Member 7 | ENSG00000131269 | ***P* < 0.001***** | | Increase | *P* = 0.637 |
|  | 25 | *COX19* | Cytochrome C Oxidase Assembly Factor COX19 | ENSG00000240230 | ***P* < 0.001***** | | Increase | *P* = 0.102 |
|  | 26 | *HEPH* | Hephaestin | ENSG00000089472 | *P* = 0.505 | | Uncertain |  |
|  | 27 | *COMMD1* | Copper Metabolism Domain Containing 1 | ENSG00000173163 | ***P* < 0.001***** | | Increase | *P* = 0.216 |
|  | 28 | *AOC2* | Amine Oxidase Copper Containing 2 | ENSG00000131480 | ***P* < 0.001***** | | Increase | *P* = 0.111 |
|  | 29 | *AOC3* | Amine Oxidase Copper Containing 3 | ENSG00000131471 | ***P* = 0.005**** | | Decline | *P* = 0.931 |
|  | 30 | *CUTC* | CutC Copper Transporter | ENSG00000119929 | ***P* < 0.001***** | | Increase | *P* = 0.223 |
| Copper metabolism disease related genes | 1 | *TMEM199* | Transmembrane Protein 199 | ENSG00000244045 | ***P* < 0.001***** | | Increase | ***P* = 0.003**** |
|  | 2 | *ATP6AP1* | ATPase H+ Transporting Accessory Protein 1 | ENSG00000071553 | ***P* < 0.001***** | | Increase | ***P* = 0.003**** |
|  | 3 | *SLC33A1* | Solute Carrier Family 33 Member 1 | ENSG00000169359 | ***P* < 0.001***** | | Increase | *P* = 0.094 |
|  | 4 | *AP1S1* | Adaptor Related Protein Complex 1 Subunit Sigma 1 | ENSG00000106367 | ***P* < 0.001***** | | Increase | *P* = 0.212 |
|  | 5 | *CCDC115* | Coiled-Coil Domain Containing 115 | ENSG00000136710 | ***P* < 0.001***** | | Increase | *P* = 0.181 |
|  | 6 | *ATP7BP1* | ATPase Copper Transporting Beta Pseudogene 1 | ENSG00000270855 | ***P* < 0.001***** | | Increase | *P* = 0.181 |
|  | 7 | *ATP8B1* | ATPase Phospholipid Transporting 8B1 | ENSG00000081923 | ***P* < 0.001***** | | Increase | *P* = 0.277 |
|  | 8 | *ABCB4* | ATP Binding Cassette Subfamily B Member 4 | ENSG00000005471 | ***P* < 0.001***** | | Decline | *P* = 0.189 |
|  | 9 | *ABCB11* | ATP Binding Cassette Subfamily B Member 11 | ENSG00000073734 | ***P* < 0.001***** | | Decline | *P* = 0.078 |
|  | 10 | *MPI* | Mannose Phosphate Isomerase | ENSG00000178802 | ***P* < 0.001***** | | Increase | *P* = 0.232 |
| Cuproptosis genes related mitochondrial respiration | 1 | *FDX1* | Ferredoxin 1 | ENSG00000137714 | ***P* = 0.003**** | | Decline | ***P* = 0.035*** |
|  | 2 | *LIPT1* | Lipoyltransferase 1 | ENSG00000144182 | ***P* < 0.001***** | | Increase | ***P* = 0.043*** |
|  | 3 | *DLAT* | Dihydrolipoamide S-Acetyltransferase | ENSG00000150768 | ***P* < 0.001***** | | Increase | ***P* = 0.004**** |
|  | 4 | *PDHA1* | Pyruvate Dehydrogenase E1 Subunit Alpha 1 | ENSG00000131828 | ***P* < 0.001***** | | Increase | ***P* = 0.011*** |
|  | 5 | *MTF1* | Metal Regulatory Transcription Factor 1 | ENSG00000188786 | ***P* < 0.001***** | | Increase | ***P* = 0.021*** |
|  | 6 | *MT-CO1* | Mitochondrially Encoded Cytochrome C Oxidase I | ENSG00000198804 | ***P* < 0.001***** | | Decline | ***P* = 0.004**** |
|  | 7 | *ACO1* | Aconitase 1 | ENSG00000122729 | ***P* < 0.001***** | | Decline | ***P* = 0.017*** |
|  | 8 | *CDKN2A* | Cyclin Dependent Kinase Inhibitor 2A | ENSG00000147889 | ***P* < 0.001***** | | Increase | ***P* = 0.004**** |
|  | 9 | *CDKN3* | Cyclin Dependent Kinase Inhibitor 3 | ENSG00000100526 | ***P* < 0.001***** | | Increase | ***P* = 0.025*** |
|  | 10 | *TPI1* | Triosephosphate Isomerase 1 | ENSG00000111669 | ***P* < 0.001***** | | Increase | ***P* < 0.001***** |
|  | 11 | *LIAS* | Lipoic Acid Synthetase | ENSG00000121897 | ***P* < 0.001***** | | Increase | *P* = 0.614 |
|  | 12 | *DLD* | Dihydrolipoamide Dehydrogenase | ENSG00000091140 | ***P* = 0.001**** | | Increase | *P* = 0.704 |
|  | 13 | *PDHB* | Pyruvate Dehydrogenase E1 Subunit Beta | ENSG00000168291 | ***P* < 0.001***** | | Increase | *P* = 0.735 |
|  | 14 | *GLS* | Glutaminase | ENSG00000115419 | ***P* < 0.001***** | | Increase | *P* = 0.072 |
|  | 15 | *NLRP3* | NLR Family Pyrin Domain Containing 3 | ENSG00000162711 | ***P* = 0.007**** | | Decline | *P* = 0.216 |
|  | 16 | *NFE2L2* | NFE2 Like BZIP Transcription Factor 2 | ENSG00000116044 | ***P* = 0.02*** | | Decline | *P* = 0.427 |
|  | 17 | *LIPT2* | Lipoyl (Octanoyl) Transferase 2 | ENSG00000175536 | ***P* < 0.001***** | | Increase | *P* = 0.464 |
|  | 18 | *GCSH* | Glycine Cleavage System Protein H | ENSG00000140905 | ***P* < 0.001***** | | Decline | *P* = 0.645 |
|  | 19 | *DLST* | Dihydrolipoamide S-Succinyltransferase | ENSG00000119689 | ***P* < 0.001***** | | Increase | *P* = 0.053 |
|  | 20 | *DBT* | Dihydrolipoamide Branched Chain Transacylase E2 | ENSG00000137992 | ***P* < 0.001***** | | Decline | *P* = 0.122 |
|  | 21 | *MT-CO2* | Mitochondrially Encoded Cytochrome C Oxidase Ⅱ | ENSG00000198712 | ***P* < 0.001***** | | Decline | *P* = 0.231 |
|  | 22 | *MT-CO3* | Mitochondrially Encoded Cytochrome C Oxidase Ⅲ | ENSG00000198938 | ***P* < 0.001***** | | Decline | *P* = 0.06 |
|  | 23 | *ACO2* | Aconitase 2 | ENSG00000100412 | ***P* < 0.001***** | | Increase | *P* = 0.449 |
|  | 24 | *SDHA* | Succinate Dehydrogenase Complex Flavoprotein Subunit A | ENSG00000073578 | ***P* < 0.001***** | | Decline | *P* = 0.751 |
|  | 25 | *SDHB* | Succinate Dehydrogenase Complex Iron Sulfur Subunit B | ENSG00000117118 | ***P* < 0.001***** | | Decline | *P* = 0.864 |
|  | 26 | *SDHC* | Succinate Dehydrogenase Complex Subunit C | ENSG00000143252 | ***P* < 0.001***** | | Increase | *P* = 0.454 |
|  | 27 | *SDHD* | Succinate Dehydrogenase Complex Subunit D | ENSG00000204370 | ***P* < 0.001***** | | Decline | *P* = 0.381 |
|  | 28 | *IDH2* | Isocitrate Dehydrogenase (NADP(+)) 2 | ENSG00000182054 | *P* = 0.098 | | Decline |  |
|  | 29 | *IDH1* | Isocitrate Dehydrogenase (NADP(+)) 1 | ENSG00000138413 | *P* = 0.622 | | Uncertain |  |
| Genes of iron-sulfur cluster related proteins | 1 | *ACP1* | Acid Phosphatase 1 | ENSG00000143727 | ***P* < 0.001***** | | Increase | ***P* < 0.001***** |
|  | 2 | *FDX2* | Ferredoxin 2 | ENSG00000267673 | ***P* < 0.001***** | | Increase | ***P* = 0.01*** |
|  | 3 | *NUBP2* | Nucleotide Binding Protein 2 | ENSG00000095906 | ***P* < 0.001***** | | Increase | ***P* = 0.041*** |
|  | 4 | *CIAPIN1* | Cytokine Induced Apoptosis Inhibitor 1 | ENSG00000005194 | ***P* < 0.001***** | | Increase | ***P* = 0.033*** |
|  | 5 | *ISCA2* | Iron-Sulfur Cluster Assembly 2 | ENSG00000165898 | ***P* < 0.001***** | | Increase | ***P* = 0.003**** |
|  | 6 | *NDOR1* | NADPH Dependent Diflavin Oxidoreductase 1 | ENSG00000188566 | ***P* < 0.001***** | | Increase | ***P* = 0.001**** |
|  | 7 | *HSPA8* | Heat Shock Protein Family A (Hsp70) Member 8 | ENSG00000109971 | ***P* < 0.001***** | | Increase | ***P* = 0.005**** |
|  | 8 | *FXN* | Frataxin | ENSG00000165060 | ***P* < 0.001***** | | Decline | *P* = 0.855 |
|  | 9 | *ISCU* | Iron-Sulfur Cluster Assembly Enzyme | ENSG00000136003 | ***P* < 0.001***** | | Increase | *P* = 0.736 |
|  | 10 | *GLRX5* | Glutaredoxin 5 | ENSG00000182512 | ***P* = 0.005**** | | Increase | *P* = 0.834 |
|  | 11 | *MT1E* | Metallothionein 1E | ENSG00000169715 | ***P* < 0.001***** | | Decline | *P* = 0.715 |
|  | 12 | *MT1F* | Metallothionein 1F | ENSG00000198417 | ***P* < 0.001***** | | Decline | *P* = 0.528 |
|  | 13 | *MT1G* | Metallothionein 1G | ENSG00000125144 | ***P* < 0.001***** | | Decline | *P* = 0.589 |
|  | 14 | *MT1X* | Metallothionein 1X | ENSG00000187193 | ***P* < 0.001***** | | Decline | *P* = 0.356 |
|  | 15 | *NFS1* | NFS1 Cysteine Desulfurase | ENSG00000244005 | ***P* < 0.001***** | | Increase | *P* = 0.226 |
|  | 16 | *NFU1* | NFU1 Iron-Sulfur Cluster Scaffold | ENSG00000169599 | ***P* < 0.001***** | | Increase | *P* = 0.141 |
|  | 17 | *FDXR* | Ferredoxin Reductase | ENSG00000161513 | ***P* < 0.001***** | | Increase | *P* = 0.927 |
|  | 18 | *DPYD* | Dihydropyrimidine Dehydrogenase | ENSG00000188641 | ***P* < 0.001***** | | Decline | *P* = 0.715 |
|  | 19 | *NUBPL* | NUBP Iron-Sulfur Cluster Assembly Factor, Mitochondrial | ENSG00000151413 | *P* = 0.542 | | Uncertain |  |
|  | 20 | *NUBP1* | NUBP Iron-Sulfur Cluster Assembly Factor 1, Cytosolic | ENSG00000103274 | ***P* < 0.001***** | | Increase | *P* = 0.804 |
|  | 21 | *HSPA9* | Heat Shock Protein Family A (Hsp70) Member 9 | ENSG00000113013 | ***P* < 0.001***** | | Increase | *P* = 0.103 |
|  | 22 | *ISCA1* | Iron-Sulfur Cluster Assembly 1 | ENSG00000135070 | ***P* < 0.001***** | | Increase | *P* = 0.487 |
|  | 23 | *IBA57* | Iron-Sulfur Cluster Assembly Factor IBA57 | ENSG00000181873 | ***P* < 0.001***** | | Increase | *P* = 0.527 |
|  | 24 | *HSCB* | HscB Mitochondrial Iron-Sulfur Cluster Cochaperone | ENSG00000100209 | ***P* < 0.001***** | | Increase | *P* = 0.99 |
|  | 25 | *CIAO1* | Cytosolic Iron-Sulfur Assembly Component 1 | ENSG00000144021 | ***P* < 0.001***** | | Increase | *P* = 0.117 |
|  | 26 | *CIAO2A* | Cytosolic Iron-Sulfur Assembly Component 2A | ENSG00000166797 | ***P* = 0.008**** | | Increase | *P* = 0.327 |
|  | 27 | *CIAO2B* | Cytosolic Iron-Sulfur Assembly Component 2B | ENSG00000166595 | ***P* < 0.001***** | | Increase | *P* = 0.164 |

Note: “*”, *P* < 0.05; “**”, *P* < 0.01; “***”, *P* < 0.001.
