# Supplementary material for: The expression of cuproptosis-related genes in hepatocellular carcinoma and their relationships with prognosis
Source: Front Oncol. 2022 Oct 14;12:992468. doi: 10.3389/fonc.2022.992468 (PMC9614267; doi:10.3389/fonc.2022.992468)
Supplement: Supplementary file 2 [file DataSheet_2.docx]

**Supplementary Table 2.** Logistic regression of gene expression and clinicopathological variables

| **Gene** | **Clinical characteristics** | **Total number** | **The Odds ratio in gene expression** | ***P*-value** |
| --- | --- | --- | --- | --- |
| *SCO2* | Clinical stage (Ⅱ vs. Ⅰ) | 284 | 1.330 (0.812 - 2.186) | 0.258 |
|  | Clinical stage (Ⅲ vs. Ⅰ) | 275 | 1.320 (0.792 - 2.206) | 0.287 |
|  | Clinical stage (Ⅳ vs. Ⅰ) | 199 | 1.148 (0.310 - 4.252) | 0.832 |
|  | Histologic grade (G2 vs. G1) | 232 | 1.355 (0.736 - 2.530) | 0.333 |
|  | Histologic grade (G3 vs. G1) | 177 | 2.088 (1.098 - 4.034) | **0.026*** |
|  | Histologic grade (G4 vs. G1) | 67 | 1.500 (0.419 - 5.386) | 0.526 |
|  | Tumor stage (T2 vs. T1) | 303 | 1.305 (0.812 - 2.104) | 0.272 |
|  | Tumor stage (T3 vs. T1) | 284 | 1.509 (0.907 - 2.527) | 0.115 |
|  | Tumor stage (T4 vs. T1) | 212 | 0.727 (0.213 - 2.256) | 0.587 |
|  | Distant metastasis (M1 vs. M0) | 300 | 1.342 (0.291 - 6.914) | 0.703 |
|  | Lymph nodes (N1 vs. N0) | 285 | 0.596 (0.120 - 2.475) | 0.484 |
| *ATP7A* | Clinical stage (Ⅱ vs. Ⅰ) | 284 | 1.648 (1.005 - 2.716) | **0.049*** |
|  | Clinical stage (Ⅲ vs. Ⅰ) | 275 | 2.084 (1.244 - 3.526) | **0.006**** |
|  | Clinical stage (Ⅳ vs. Ⅰ) | 199 | 2.044 (0.565 - 8.217) | 0.280 |
|  | Histologic grade (G2 vs. G1) | 232 | 1.324 (0.720 - 2.474) | 0.370 |
|  | Histologic grade (G3 vs. G1) | 177 | 2.235 (1.174 - 4.323) | **0.015*** |
|  | Histologic grade (G4 vs. G1) | 67 | 1.071 (0.285 - 3.789) | 0.915 |
|  | Tumor stage (T2 vs. T1) | 303 | 1.906 (1.182 - 3.094) | **0.009**** |
|  | Tumor stage (T3 vs. T1) | 284 | 1.997 (1.196 - 3.363) | **0.009**** |
|  | Tumor stage (T4 vs. T1) | 212 | 1.631 (0.523 - 5.233) | 0.395 |
|  | Distant metastasis (M1 vs. M0) | 300 | 0.745 (0.145 - 3.435) | 0.703 |
|  | Lymph nodes (N1 vs. N0) | 285 | 7.363 (1.287 - 138.564) | 0.063 |
| *SLC25A3* | Clinical stage (Ⅱ vs. Ⅰ) | 284 | 0.910 (0.554 - 1.490) | 0.707 |
|  | Clinical stage (Ⅲ vs. Ⅰ) | 275 | 1.396 (0.837 - 2.343) | 0.203 |
|  | Clinical stage (Ⅳ vs. Ⅰ) | 199 | 1.054 (0.285 - 3.905) | 0.935 |
|  | Histologic grade (G2 vs. G1) | 232 | 1.041 (0.567 - 1.928) | 0.897 |
|  | Histologic grade (G3 vs. G1) | 177 | 1.798 (0.948 - 3.445) | 0.074 |
|  | Histologic grade (G4 vs. G1) | 67 | 3.875 (1.029 - 18.915) | 0.060 |
|  | Tumor stage (T2 vs. T1) | 303 | 0.993 (0.617 - 1.597) | 0.978 |
|  | Tumor stage (T3 vs. T1) | 284 | 1.392 (0.837 - 2.330) | 0.205 |
|  | Tumor stage (T4 vs. T1) | 212 | 0.920 (0.287 - 2.863) | 0.884 |
|  | Distant metastasis (M1 vs. M0) | 300 | 0.745 (0.145 - 3.435) | 0.703 |
|  | Lymph nodes (N1 vs. N0) | 285 | 1.703 (0.410 - 8.432) | 0.472 |
| *AOC1* | Clinical stage (Ⅱ vs. Ⅰ) | 284 | 0.969 (0.591 - 1.588) | 0.901 |
|  | Clinical stage (Ⅲ vs. Ⅰ) | 275 | 1.426 (0.855 - 2.393) | 0.175 |
|  | Clinical stage (Ⅳ vs. Ⅰ) | 199 | 1.077 (0.291 - 3.989) | 0.909 |
|  | Histologic grade (G2 vs. G1) | 232 | 1.138 (0.618 - 2.089) | 0.676 |
|  | Histologic grade (G3 vs. G1) | 177 | 0.644 (0.338 - 1.220) | 0.178 |
|  | Histologic grade (G4 vs. G1) | 67 | 0.448 (0.109 - 1.599) | 0.231 |
|  | Tumor stage (T2 vs. T1) | 303 | 1.014 (0.630 - 1.630) | 0.955 |
|  | Tumor stage (T3 vs. T1) | 284 | 1.232 (0.741 - 2.053) | 0.422 |
|  | Tumor stage (T4 vs. T1) | 212 | 3.649 (1.079 - 16.635) | 0.055 |
|  | Distant metastasis (M1 vs. M0) | 300 | 1.342 (0.291 - 6.914) | 0.703 |
|  | Lymph nodes (N1 vs. N0) | 285 | 1.007 (0.234 - 4.336) | 0.992 |
| *COA6* | Clinical stage (Ⅱ vs. Ⅰ) | 284 | 1.915 (1.165 - 3.172) | **0.011*** |
|  | Clinical stage (Ⅲ vs. Ⅰ) | 275 | 1.533 (0.920 - 2.567) | 0.102 |
|  | Clinical stage (Ⅳ vs. Ⅰ) | 199 | 3.111 (0.837 - 14.775) | 0.108 |
|  | Histologic grade (G2 vs. G1) | 232 | 1.023 (0.556 - 1.904) | 0.942 |
|  | Histologic grade (G3 vs. G1) | 177 | 2.466 (1.293 - 4.773) | **0.007**** |
|  | Histologic grade (G4 vs. G1) | 67 | 1.948 (0.553 - 7.321) | 0.302 |
|  | Tumor stage (T2 vs. T1) | 303 | 1.688 (1.048 - 2.733) | **0.032*** |
|  | Tumor stage (T3 vs. T1) | 284 | 1.752 (1.052 - 2.942) | **0.032*** |
|  | Tumor stage (T4 vs. T1) | 212 | 0.805 (0.236 - 2.498) | 0.711 |
|  | Distant metastasis (M1 vs. M0) | 300 | 2.552 (0.541 - 18.015) | 0.267 |
|  | Lymph nodes (N1 vs. N0) | 285 | 1.703 (0.410 - 8.432) | 0.472 |
| *TMEM199* | Clinical stage (Ⅱ vs. Ⅰ) | 284 | 1.719 (1.047 - 2.841) | **0.033*** |
|  | Clinical stage (Ⅲ vs. Ⅰ) | 275 | 1.438 (0.863 - 2.405) | 0.165 |
|  | Clinical stage (Ⅳ vs. Ⅰ) | 199 | 1.250 (0.337 - 4.632) | 0.731 |
|  | Histologic grade (G2 vs. G1) | 232 | 1.182 (0.642 - 2.208) | 0.595 |
|  | Histologic grade (G3 vs. G1) | 177 | 2.235 (1.174 - 4.323) | **0.015*** |
|  | Histologic grade (G4 vs. G1) | 67 | 7.500 (1.766 - 51.944) | **0.014*** |
|  | Tumor stage (T2 vs. T1) | 303 | 1.755 (1.090 - 2.846) | **0.021*** |
|  | Tumor stage (T3 vs. T1) | 284 | 1.592 (0.957 - 2.665) | 0.074 |
|  | Tumor stage (T4 vs. T1) | 212 | 1.103 (0.344 - 3.437) | 0.864 |
|  | Distant metastasis (M1 vs. M0) | 300 | 1.342 (0.291 - 6.914) | 0.703 |
|  | Lymph nodes (N1 vs. N0) | 285 | 1.007 (0.234 - 4.336) | 0.992 |
| *ATP6AP1* | Clinical stage (Ⅱ vs. Ⅰ) | 284 | 1.223 (0.746 - 2.007) | 0.424 |
|  | Clinical stage (Ⅲ vs. Ⅰ) | 275 | 1.443 (0.866 - 2.417) | 0.160 |
|  | Clinical stage (Ⅳ vs. Ⅰ) | 199 | 10.779 (1.969 - 200.755) | **0.025*** |
|  | Histologic grade (G2 vs. G1) | 232 | 1.141 (0.622 - 2.111) | 0.672 |
|  | Histologic grade (G3 vs. G1) | 177 | 1.573 (0.831 - 3.008) | 0.166 |
|  | Histologic grade (G4 vs. G1) | 67 | 3.875 (1.029 - 18.915) | 0.060 |
|  | Tumor stage (T2 vs. T1) | 303 | 1.359 (0.845 - 2.192) | 0.206 |
|  | Tumor stage (T3 vs. T1) | 284 | 1.571 (0.944 - 2.632) | 0.083 |
|  | Tumor stage (T4 vs. T1) | 212 | 1.413 (0.454 - 4.532) | 0.547 |
|  | Distant metastasis (M1 vs. M0) | 300 | 16417511 (7.76933E-29 - Na) | 0.985 |
|  | Lymph nodes (N1 vs. N0) | 285 | 3.110 (0.703 - 21.480) | 0.169 |
| *FDX1* | Clinical stage (Ⅱ vs. Ⅰ) | 284 | 0.533 (0.323 - 0.875) | **0.013*** |
|  | Clinical stage (Ⅲ vs. Ⅰ) | 275 | 0.419 (0.246 - 0.703) | **0.001**** |
|  | Clinical stage (Ⅳ vs. Ⅰ) | 199 | 0.168 (0.025 - 0.693) | **0.027*** |
|  | Histologic grade (G2 vs. G1) | 232 | 0.626 (0.328 - 1.163) | 0.144 |
|  | Histologic grade (G3 vs. G1) | 177 | 0.331 (0.168 - 0.636) | **0.001**** |
|  | Histologic grade (G4 vs. G1) | 67 | 0.264 (0.063 - 0.950) | **0.048*** |
|  | Tumor stage (T2 vs. T1) | 303 | 0.546 (0.337 - 0.879) | **0.013*** |
|  | Tumor stage (T3 vs. T1) | 284 | 0.454 (0.268 - 0.760) | **0.003**** |
|  | Tumor stage (T4 vs. T1) | 212 | 0.613 (0.191 - 1.911) | 0.395 |
|  | Distant metastasis (M1 vs. M0) | 300 | 0.392 (0.056 - 1.850) | 0.267 |
|  | Lymph nodes (N1 vs. N0) | 285 | 0.138 (0.007 - 0.789) | 0.065 |
| *LIPT1* | Clinical stage (Ⅱ vs. Ⅰ) | 284 | 1.359 (0.829 - 2.235) | 0.225 |
|  | Clinical stage (Ⅲ vs. Ⅰ) | 275 | 1.024 (0.613 - 1.706) | 0.928 |
|  | Clinical stage (Ⅳ vs. Ⅰ) | 199 | 4.494 (1.091 - 30.303) | 0.061 |
|  | Histologic grade (G2 vs. G1) | 232 | 1.581 (0.854 - 2.988) | 0.150 |
|  | Histologic grade (G3 vs. G1) | 177 | 2.436 (1.274 - 4.760) | **0.008**** |
|  | Histologic grade (G4 vs. G1) | 67 | 3.500 (0.975 - 14.509) | 0.063 |
|  | Tumor stage (T2 vs. T1) | 303 | 1.437 (0.893 - 2.322) | 0.136 |
|  | Tumor stage (T3 vs. T1) | 284 | 1.167 (0.702 - 1.943) | 0.552 |
|  | Tumor stage (T4 vs. T1) | 212 | 0.977 (0.305 - 3.042) | 0.968 |
|  | Distant metastasis (M1 vs. M0) | 300 | 2.552 (0.541 - 18.015) | 0.267 |
|  | Lymph nodes (N1 vs. N0) | 285 | 7.363 (1.287 - 138.564) | 0.063 |
| *DLAT* | Clinical stage (Ⅱ vs. Ⅰ) | 284 | 1.124 (0.686 - 1.842) | 0.643 |
|  | Clinical stage (Ⅲ vs. Ⅰ) | 275 | 1.450 (0.870 - 2.430) | 0.156 |
|  | Clinical stage (Ⅳ vs. Ⅰ) | 199 | 2.678 (0.721 - 12.713) | 0.163 |
|  | Histologic grade (G2 vs. G1) | 232 | 1.834 (0.994 - 3.445) | 0.055 |
|  | Histologic grade (G3 vs. G1) | 177 | 1.467 (0.771 - 2.839) | 0.247 |
|  | Histologic grade (G4 vs. G1) | 67 | 8.095 (1.903 - 56.131) | **0.011*** |
|  | Tumor stage (T2 vs. T1) | 303 | 1.282 (0.797 - 2.065) | 0.306 |
|  | Tumor stage (T3 vs. T1) | 284 | 1.400 (0.842 - 2.338) | 0.196 |
|  | Tumor stage (T4 vs. T1) | 212 | 2.670 (0.840 - 10.118) | 0.112 |
|  | Distant metastasis (M1 vs. M0) | 300 | 2.552 (0.541 - 18.015) | 0.267 |
|  | Lymph nodes (N1 vs. N0) | 285 | 7.363 (1.287 - 138.564) | 0.063 |
| *PDHA1* | Clinical stage (Ⅱ vs. Ⅰ) | 284 | 0.969 (0.591 - 1.587) | 0.901 |
|  | Clinical stage (Ⅲ vs. Ⅰ) | 275 | 1.244 (0.747 - 2.081) | 0.403 |
|  | Clinical stage (Ⅳ vs. Ⅰ) | 199 | 0.688 (0.171 - 2.486) | 0.572 |
|  | Histologic grade (G2 vs. G1) | 232 | 0.916 (0.499 - 1.682) | 0.776 |
|  | Histologic grade (G3 vs. G1) | 177 | 1.144 (0.605 - 2.170) | 0.678 |
|  | Histologic grade (G4 vs. G1) | 67 | 3.111 (0.827 - 15.161) | 0.115 |
|  | Tumor stage (T2 vs. T1) | 303 | 1.093 (0.680 - 1.758) | 0.714 |
|  | Tumor stage (T3 vs. T1) | 284 | 1.128 (0.679 - 1.879) | 0.641 |
|  | Tumor stage (T4 vs. T1) | 212 | 0.901 (0.281 - 2.806) | 0.856 |
|  | Distant metastasis (M1 vs. M0) | 300 | 0.392 (0.056 - 1.850) | 0.267 |
|  | Lymph nodes (N1 vs. N0) | 285 | 1.703 (0.410 - 8.432) | 0.472 |
| *MTF1* | Clinical stage (Ⅱ vs. Ⅰ) | 284 | 1.275 (0.779 - 2.094) | 0.335 |
|  | Clinical stage (Ⅲ vs. Ⅰ) | 275 | 1.320 (0.792 - 2.206) | 0.287 |
|  | Clinical stage (Ⅳ vs. Ⅰ) | 199 | 1.722 (0.477 - 6.918) | 0.412 |
|  | Histologic grade (G2 vs. G1) | 232 | 0.932 (0.507 - 1.710) | 0.820 |
|  | Histologic grade (G3 vs. G1) | 177 | 0.996 (0.526 - 1.886) | 0.991 |
|  | Histologic grade (G4 vs. G1) | 67 | 0.964 (0.270 - 3.441) | 0.954 |
|  | Tumor stage (T2 vs. T1) | 303 | 1.439 (0.895 - 2.324) | 0.134 |
|  | Tumor stage (T3 vs. T1) | 284 | 1.274 (0.766 - 2.123) | 0.351 |
|  | Tumor stage (T4 vs. T1) | 212 | 1.899 (0.612 - 6.472) | 0.275 |
|  | Distant metastasis (M1 vs. M0) | 300 | 0.745 (0.145 - 3.435) | 0.703 |
|  | Lymph nodes (N1 vs. N0) | 285 | 3.110 (0.703 - 21.480) | 0.169 |
| *MT-CO1* | Clinical stage (Ⅱ vs. Ⅰ) | 284 | 0.720 (0.438 - 1.180) | 0.194 |
|  | Clinical stage (Ⅲ vs. Ⅰ) | 275 | 0.693 (0.414 - 1.155) | 0.160 |
|  | Clinical stage (Ⅳ vs. Ⅰ) | 199 | 0.557 (0.139 - 2.011) | 0.376 |
|  | Histologic grade (G2 vs. G1) | 232 | 0.801 (0.433 - 1.469) | 0.475 |
|  | Histologic grade (G3 vs. G1) | 177 | 0.657 (0.344 - 1.244) | 0.199 |
|  | Histologic grade (G4 vs. G1) | 67 | 0.774 (0.216 - 2.765) | 0.688 |
|  | Tumor stage (T2 vs. T1) | 303 | 0.751 (0.466 - 1.207) | 0.237 |
|  | Tumor stage (T3 vs. T1) | 284 | 0.714 (0.428 - 1.188) | 0.196 |
|  | Tumor stage (T4 vs. T1) | 212 | 0.374 (0.099 - 1.191) | 0.112 |
|  | Distant metastasis (M1 vs. M0) | 300 | 0.392 (0.056 - 1.850) | 0.267 |
|  | Lymph nodes (N1 vs. N0) | 285 | 0.596 (0.120 - 2.475) | 0.484 |
| *ACO1* | Clinical stage (Ⅱ vs. Ⅰ) | 284 | 0.801 (0.488 - 1.312) | 0.378 |
|  | Clinical stage (Ⅲ vs. Ⅰ) | 275 | 0.811 (0.486 - 1.351) | 0.421 |
|  | Clinical stage (Ⅳ vs. Ⅰ) | 199 | 0.593 (0.148 - 2.144) | 0.430 |
|  | Histologic grade (G2 vs. G1) | 232 | 0.449 (0.234 - 0.839) | **0.014*** |
|  | Histologic grade (G3 vs. G1) | 177 | 0.427 (0.216 - 0.821) | **0.012*** |
|  | Histologic grade (G4 vs. G1) | 67 | 0.243 (0.058 - 0.878) | **0.037*** |
|  | Tumor stage (T2 vs. T1) | 303 | 0.722 (0.448 - 1.161) | 0.180 |
|  | Tumor stage (T3 vs. T1) | 284 | 0.649 (0.388 - 1.081) | 0.098 |
|  | Tumor stage (T4 vs. T1) | 212 | 0.983 (0.316 - 3.152) | 0.976 |
|  | Distant metastasis (M1 vs. M0) | 300 | 0.745 (0.145 - 3.435) | 0.703 |
|  | Lymph nodes (N1 vs. N0) | 285 | 0.596 (0.120 - 2.475) | 0.484 |
| *ACP1* | Clinical stage (Ⅱ vs. Ⅰ) | 284 | 1.275 (0.779 - 2.094) | 0.335 |
|  | Clinical stage (Ⅲ vs. Ⅰ) | 275 | 1.383 (0.830 - 2.315) | 0.214 |
|  | Clinical stage (Ⅳ vs. Ⅰ) | 199 | 1.148 (0.310 - 4.252) | 0.832 |
|  | Histologic grade (G2 vs. G1) | 232 | 1.335 (0.723 - 2.509) | 0.360 |
|  | Histologic grade (G3 vs. G1) | 177 | 2.496 (1.307 - 4.858) | **0.006**** |
|  | Histologic grade (G4 vs. G1) | 67 | 3.238 (0.904 - 13.394) | 0.081 |
|  | Tumor stage (T2 vs. T1) | 303 | 1.332 (0.829 - 2.147) | 0.237 |
|  | Tumor stage (T3 vs. T1) | 284 | 1.335 (0.803 - 2.227) | 0.266 |
|  | Tumor stage (T4 vs. T1) | 212 | 2.670 (0.840 - 10.118) | 0.112 |
|  | Distant metastasis (M1 vs. M0) | 300 | 0.745 (0.145 - 3.435) | 0.703 |
|  | Lymph nodes (N1 vs. N0) | 285 | 1.703 (0.410 - 8.432) | 0.472 |
| *FDX2* | Clinical stage (Ⅱ vs. Ⅰ) | 284 | 1.275 (0.778 - 2.095) | 0.335 |
|  | Clinical stage (Ⅲ vs. Ⅰ) | 275 | 1.050 (0.630 - 1.750) | 0.851 |
|  | Clinical stage (Ⅳ vs. Ⅰ) | 199 | 2.567 (0.691 - 12.183) | 0.181 |
|  | Histologic grade (G2 vs. G1) | 232 | 1.418 (0.771 - 2.647) | 0.266 |
|  | Histologic grade (G3 vs. G1) | 177 | 1.768 (0.931 - 3.406) | 0.084 |
|  | Histologic grade (G4 vs. G1) | 67 | 4.500 (1.193 - 22.016) | **0.037*** |
|  | Tumor stage (T2 vs. T1) | 303 | 1.382 (0.860 - 2.231) | 0.183 |
|  | Tumor stage (T3 vs. T1) | 284 | 1.223 (0.736 - 2.038) | 0.437 |
|  | Tumor stage (T4 vs. T1) | 212 | 0.977 (0.305 - 3.042) | 0.968 |
|  | Distant metastasis (M1 vs. M0) | 300 | 2.552 (0.541 - 18.015) | 0.267 |
|  | Lymph nodes (N1 vs. N0) | 285 | 1.007 (0.234 - 4.336) | 0.992 |
| *NUBP2* | Clinical stage (Ⅱ vs. Ⅰ) | 284 | 1.302 (0.795 - 2.140) | 0.295 |
|  | Clinical stage (Ⅲ vs. Ⅰ) | 275 | 1.177 (0.706 - 1.964) | 0.531 |
|  | Clinical stage (Ⅳ vs. Ⅰ) | 199 | 1.685 (0.467 - 6.772) | 0.430 |
|  | Histologic grade (G2 vs. G1) | 232 | 1.109 (0.605 - 2.046) | 0.739 |
|  | Histologic grade (G3 vs. G1) | 177 | 1.511 (0.798 - 2.882) | 0.206 |
|  | Histologic grade (G4 vs. G1) | 67 | 0.857 (0.229 - 3.017) | 0.811 |
|  | Tumor stage (T2 vs. T1) | 303 | 1.206 (0.751 - 1.943) | 0.439 |
|  | Tumor stage (T3 vs. T1) | 284 | 1.257 (0.756 - 2.095) | 0.379 |
|  | Tumor stage (T4 vs. T1) | 212 | 1.303 (0.419 - 4.179) | 0.645 |
|  | Distant metastasis (M1 vs. M0) | 300 | 2.552 (0.541 - 18.015) | 0.267 |
|  | Lymph nodes (N1 vs. N0) | 285 | 1.703 (0.410 - 8.432) | 0.472 |
| *CIAPIN1* | Clinical stage (Ⅱ vs. Ⅰ) | 284 | 1.054 (0.643 - 1.728) | 0.833 |
|  | Clinical stage (Ⅲ vs. Ⅰ) | 275 | 1.182 (0.710 - 1.973) | 0.521 |
|  | Clinical stage (Ⅳ vs. Ⅰ) | 199 | 2.513 (0.677 - 11.928) | 0.191 |
|  | Histologic grade (G2 vs. G1) | 232 | 1.336 (0.729 - 2.474) | 0.351 |
|  | Histologic grade (G3 vs. G1) | 177 | 1.292 (0.682 - 2.466) | 0.433 |
|  | Histologic grade (G4 vs. G1) | 67 | 2.583 (0.724 - 10.637) | 0.157 |
|  | Tumor stage (T2 vs. T1) | 303 | 1.138 (0.708 - 1.831) | 0.594 |
|  | Tumor stage (T3 vs. T1) | 284 | 1.121 (0.674 - 1.866) | 0.660 |
|  | Tumor stage (T4 vs. T1) | 212 | 2.463 (0.775 - 9.332) | 0.144 |
|  | Distant metastasis (M1 vs. M0) | 300 | 2.552 (0.541 - 18.015) | 0.267 |
|  | Lymph nodes (N1 vs. N0) | 285 | 1.703 (0.410 - 8.432) | 0.472 |
| *ISCA2* | Clinical stage (Ⅱ vs. Ⅰ) | 284 | 1.223 (0.746 - 2.007) | 0.424 |
|  | Clinical stage (Ⅲ vs. Ⅰ) | 275 | 1.586 (0.951 - 2.663) | 0.079 |
|  | Clinical stage (Ⅳ vs. Ⅰ) | 199 | 2.795 (0.752 - 13.267) | 0.145 |
|  | Histologic grade (G2 vs. G1) | 232 | 1.167 (0.636 - 2.160) | 0.620 |
|  | Histologic grade (G3 vs. G1) | 177 | 1.924 (1.014 - 3.692) | **0.046*** |
|  | Histologic grade (G4 vs. G1) | 67 | 0.258 (0.037 - 1.097) | **0.099*** |
|  | Tumor stage (T2 vs. T1) | 303 | 1.359 (0.845 - 2.192) | 0.206 |
|  | Tumor stage (T3 vs. T1) | 284 | 1.300 (0.781 - 2.167) | 0.313 |
|  | Tumor stage (T4 vs. T1) | 212 | 6.661 (1.732 - 43.769) | **0.015*** |
|  | Distant metastasis (M1 vs. M0) | 300 | 2.552 (0.541 - 18.015) | 0.267 |
|  | Lymph nodes (N1 vs. N0) | 285 | 1.703 (0.410 - 8.432) | 0.472 |
| *NDOR1* | Clinical stage (Ⅱ vs. Ⅰ) | 284 | 0.969 (0.591 - 1.587) | 0.901 |
|  | Clinical stage (Ⅲ vs. Ⅰ) | 275 | 1.244 (0.747 - 2.081) | 0.403 |
|  | Clinical stage (Ⅳ vs. Ⅰ) | 199 | 0.688 (0.171 - 2.486) | 0.572 |
|  | Histologic grade (G2 vs. G1) | 232 | 1.335 (0.723 - 2.509) | 0.360 |
|  | Histologic grade (G3 vs. G1) | 177 | 2.584 (1.352 - 5.033) | **0.005**** |
|  | Histologic grade (G4 vs. G1) | 67 | 2.267 (0.642 - 8.551) | 0.207 |
|  | Tumor stage (T2 vs. T1) | 303 | 0.900 (0.559 - 1.447) | 0.663 |
|  | Tumor stage (T3 vs. T1) | 284 | 1.310 (0.788 - 2.193) | 0.300 |
|  | Tumor stage (T4 vs. T1) | 212 | 0.449 (0.119 - 1.427) | 0.195 |
|  | Distant metastasis (M1 vs. M0) | 300 | 0.745 (0.145 - 3.435) | 0.703 |
|  | Lymph nodes (N1 vs. N0) | 285 | 3.110 (0.703 - 21.480) | 0.169 |

Note: ACO1, aconitase 1; ACP1, acid phosphatase 1; AOC1, amine oxidase copper containing 1; ATP6AP1, ATPase H+ transporting accessory protein 1; ATP7A, copper-transporting p-type adenosine triphosphatase 1; CIAPIN1, cytokine induced apoptosis inhibitor 1; COA6, cytochrome c oxidase assembly factor 6; DLAT, dihydrolipoamide S-acetyltransferase; FDX1, ferredoxin 1; FDX2, ferredoxin 2; ISCA2, iron-sulfur cluster assembly 2; LIPT1, lipoyltransferase 1; MT-CO1, mitochondrially encoded cytochrome c oxidase I; MTF1, metal regulatory transcription factor 1; NDOR1, NADPH dependent diflavin oxidoreductase 1; NUBP2, nucleotide binding protein 2; PDHA1, pyruvate dehydrogenase E1 subunit alpha 1; SCO2, synthesis of cytochrome c oxidase 2; SLC25A3, solute carrier family 25 member 3; TMEM199, transmembrane protein 199; “*”, *P* < 0.05; “**”, *P* < 0.01; “***”, *P* < 0.001.
