# Supplementary material for: The expression of cuproptosis-related genes in hepatocellular carcinoma and their relationships with prognosis
Source: Front Oncol. 2022 Oct 14;12:992468. doi: 10.3389/fonc.2022.992468 (PMC9614267; doi:10.3389/fonc.2022.992468)
Supplement: Supplementary file 3 [file Image_1.pdf]

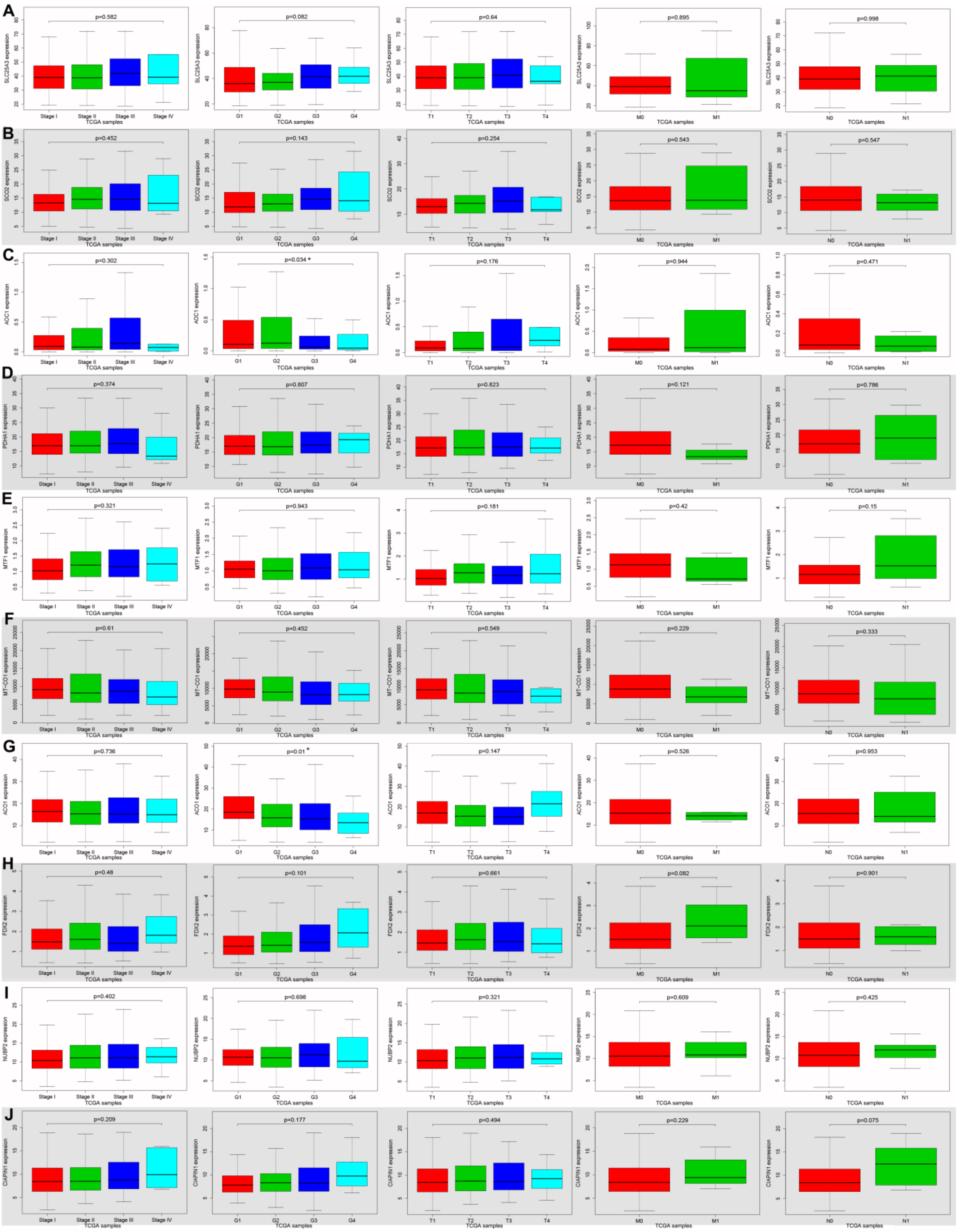

Supplementary Figure 1. The correlation between ten cuproptosis-related gene expression levels and various clinicopathological features in hepatocellular carcinoma (HCC) patients using Wilcoxon signed-rank test or Kruskal-Wallis test from the TCGA database.

The correlation between various clinicopathological features and cuproptosis-related genes expression levels in HCCs, including *SLC25A3* **(A)**, *SCO2* **(B)**, *AOC1* **(C)**, *PDHA1* **(D)**, *MTF1* **(E)**, MT-*CO1* **(F)**, *ACO1* **(G)**, *FDX2* **(H)**, *NUBP2* **(I)**, and *CIAPIN1* **(J)**. (Note: ACO1, aconitase 1; AOC1, amine oxidase copper containing 1; CIAPIN1, cytokine induced apoptosis inhibitor 1; FDX2, ferredoxin 2; G, histologic grade; HCC, hepatocellular carcinoma; M, distant metastasis ; MT-CO1, mitochondrially encoded cytochrome c oxidase I; MTF1, metal regulatory transcription factor 1; N, lymph node metastasis; NUBP2, nucleotide binding protein 2; PDHA1, pyruvate dehydrogenase E1 subunit alpha 1; SCO2, synthesis of cytochrome c oxidase 2; SLC25A3, solute carrier family 25 member 3; Stage, clinical stage; T, tumor stage; TCGA, The Cancer Genome Atlas; "*", $P < 0.05$; "**", $P < 0.01$; "***", $P < 0.001$.)

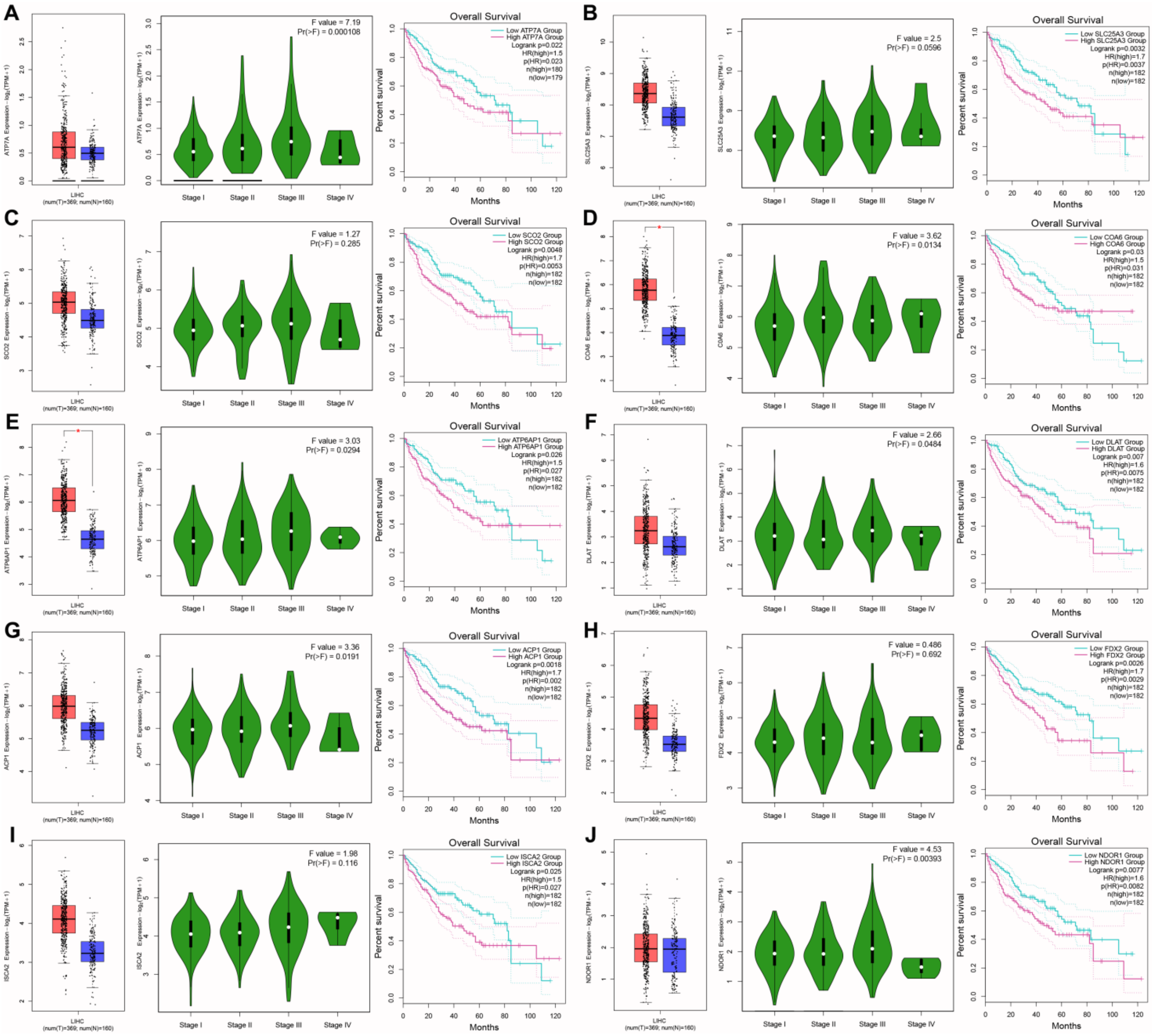

Supplementary Figure 2. The test about mRNA expressions of ten cuproptosis-related genes in the boxplot, clinical stage, and overall survival in hepatocellular carcinoma (HCC) by GEPIA2 database.

The cuproptosis-related gene including *ATP7A* **(A)**, *SLC25A3* **(B)**, *SCO2* **(C)**, *COA6* **(D)**, *ATP6AP1* **(E)**, *DLAT* **(F)**, *ACP1* **(G)**, *FDX2* **(H)**, *ISCA2* **(I)**, and *NDOR1* **(J)**. (Note: ACP1, acid phosphatase 1; ATP6AP1, ATPase H+ transporting accessory protein 1; ATP7A, copper-transporting p-type adenosine triphosphatase 1; COA6, cytochrome c oxidase assembly factor 6; DLAT, dihydrolipoamide S-acetyltransferase; FDX2, ferredoxin 2; HCC, hepatocellular carcinoma; ISCA2, iron-sulfur cluster assembly 2; NDOR1, NADPH dependent diflavin oxidoreductase 1; SCO2, synthesis of cytochrome c oxidase 2; SLC25A3, solute carrier family 25 member 3; GEPIA2, Gene Expression Profiling Interactive Analysis 2.)

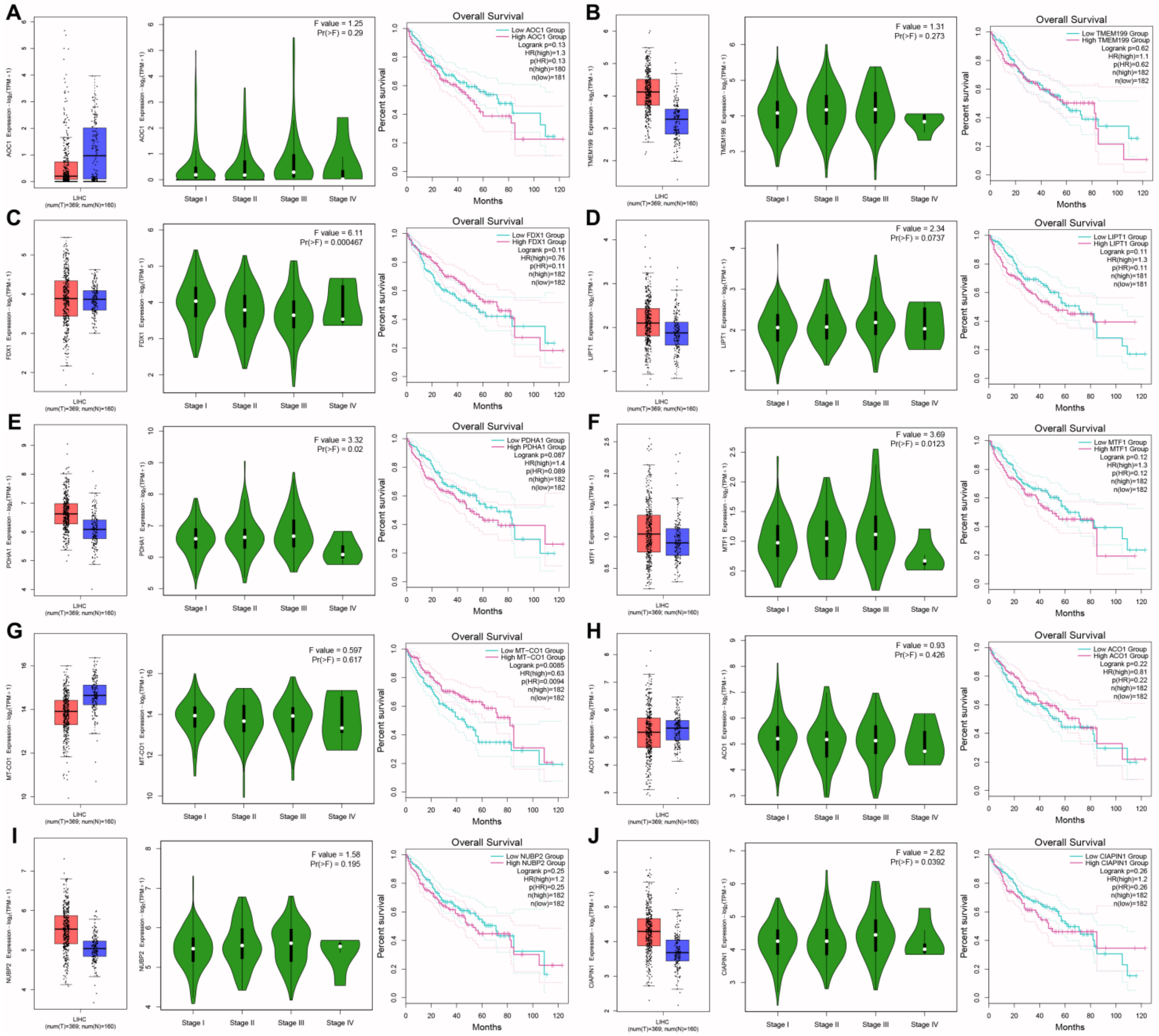

Supplementary Figure 3. The test about mRNA expressions of other ten cuproptosis-related genes in the boxplot, clinical sage, and overall survival in hepatocellular carcinoma (HCC) by GEPIA2 database.

The cuproptosis-related gene including *AOC1* **(A)**, *TMEM199* **(B)**, *FDX1* **(C)**, *LIPT1* **(D)**, *PDHA1* **(E)**, *MTF1* **(F)**, MT-*CO1* **(G)**, *ACO1* **(H)**, *NUBP2* **(I)**,and *CIAPIN1* **(J)**. (Note: ACO1, aconitase 1; AOC1, amine oxidase copper containing 1; CIAPIN1, cytokine induced apoptosis inhibitor 1; FDX1, ferredoxin 1; HCC, hepatocellular carcinoma; LIPT1, lipoyltransferase 1; MT-CO1, mitochondrially encoded cytochrome c oxidase I; MTF1, metal regulatory transcription factor 1; NUBP2, nucleotide binding protein 2; PDHA1, pyruvate dehydrogenase E1 subunit alpha 1; GEPIA2, Gene Expression Profiling Interactive Analysis 2; TMEM199, transmembrane protein 199.)

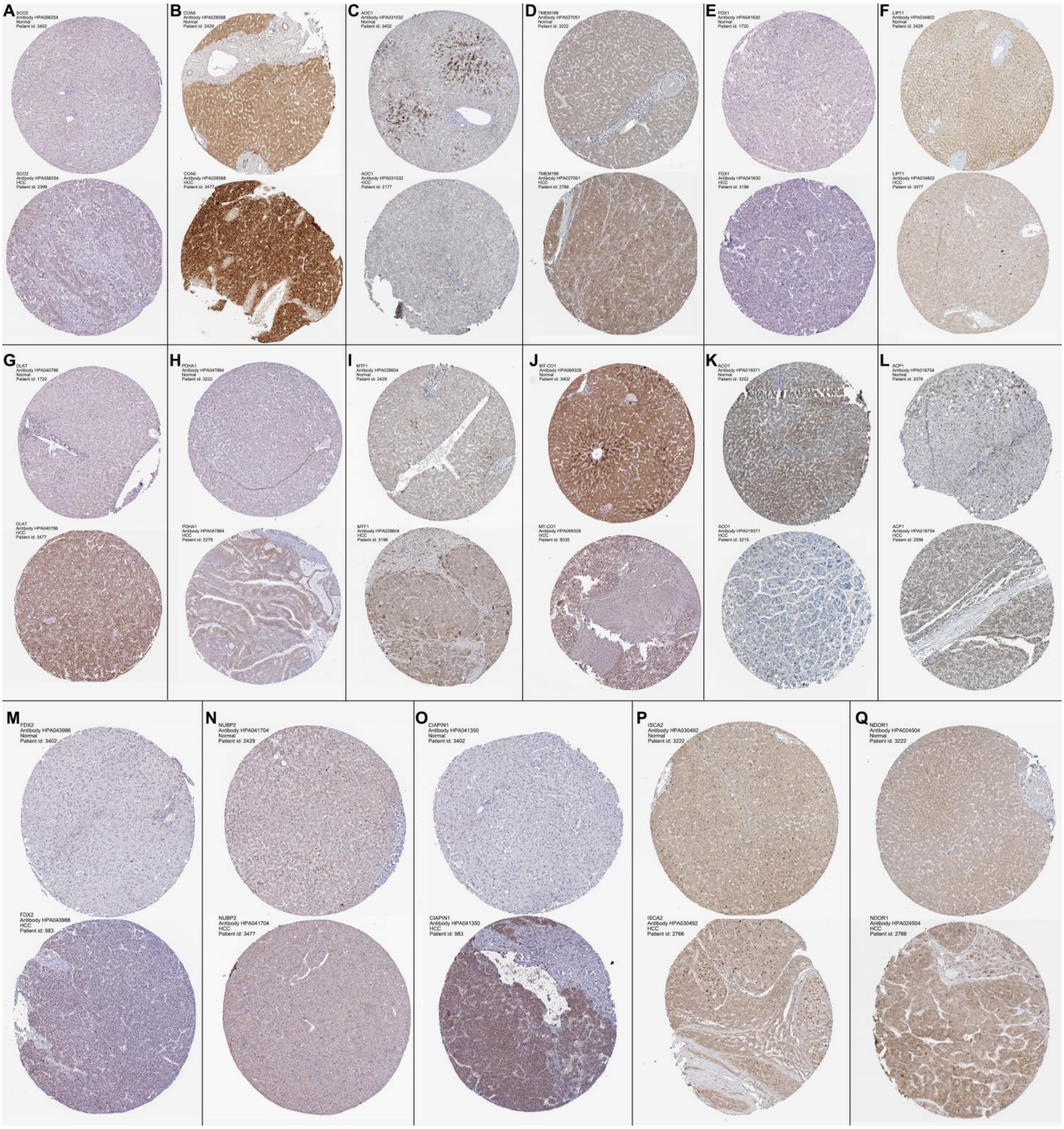

Supplementary Figure 4. The protein expression of cuproptosis-related genes between HCC and normal liver tissues in the Human Protein Atlas (HPA) database.

The protein expression of cuproptosis-related genes using the same antibody in each HCC patient and normal liver sample from HPA database, including SCO2 **(A)**, COA6 **(B)**, AOC1 **(C)**, TMEM199 **(D)**, FDX1 **(E)**, LIPT1 **(F)**, DLAT **(G)**, PDHA1 **(H)**, MTF1 **(I)**, MT-CO1 **(J)**, ACO1 **(K)**, ACP1 **(L)**, FDX2 **(M)**, NUBP2 **(N)**, CIAPIN1 **(O)**, ISCA2 **(P)**, and NDOR1 **(Q)**. (Note: ACO1, aconitase 1; ACP1, acid phosphatase 1; AOC1, amine oxidase copper containing 1; CIAPIN1, cytokine induced apoptosis inhibitor 1; COA6, cytochrome c oxidase assembly factor 6; DLAT, dihydrolipoamide S-acetyltransferase; FDX1, ferredoxin 1; FDX2, ferredoxin 2; HCC, hepatocellular carcinoma; HPA, Human Protein Atlas; ISCA2, iron-sulfur cluster assembly 2; LIPT1, lipoyltransferase 1; MT-CO1, mitochondrially encoded cytochrome c oxidase I; MTF1, metal regulatory transcription factor 1; NDOR1, NADPH dependent diflavin oxidoreductase 1; NUBP2, nucleotide binding protein 2; PDHA1, pyruvate dehydrogenase E1 subunit alpha 1; SCO2, synthesis of cytochrome c oxidase 2; TMEM199, transmembrane protein 199.)
